# Soluble phospho-tau from Alzheimer’s disease hippocampus drives microglial degeneration

**DOI:** 10.1007/s00401-016-1630-5

**Published:** 2016-10-14

**Authors:** Elisabeth Sanchez-Mejias, Victoria Navarro, Sebastian Jimenez, Maria Sanchez-Mico, Raquel Sanchez-Varo, Cristina Nuñez-Diaz, Laura Trujillo-Estrada, Jose Carlos Davila, Marisa Vizuete, Antonia Gutierrez, Javier Vitorica

**Affiliations:** 1 Departamento Biologia Celular, Genetica y Fisiologia, Facultad de Ciencias, Instituto de Biomedicina de Malaga (IBIMA), Universidad de Malaga, Campus de Teatinos s/n, 29071 Malaga, Spain; 2 Departamento Bioquimica y Biologia Molecular, Facultad de Farmacia, Universidad de Sevilla, Sevilla, Spain; 3 Instituto de Biomedicina de Sevilla (IBiS)-Hospital Universitario Virgen del Rocío/CSIC/Universidad de Sevilla, C/Prof. Garcia Gonzalez 2, 41012 Sevilla, Spain; 4 Centro de Investigacion Biomedica en Red sobre Enfermedades Neurodegenerativas (CIBERNED), Madrid, Spain

**Keywords:** Alzheimer, Microglia, Pathology, Human brain, Hippocampus

## Abstract

The role of microglial cells in the development and progression of Alzheimer’s disease (AD) has not been elucidated. Here, we demonstrated the existence of a weak microglial response in human AD hippocampus which is in contrast to the massive microglial activation observed in APP-based models. Most importantly, microglial cells displayed a prominent degenerative profile (dentate gyrus > CA3 > CA1 > parahippocampal gyrus), including fragmented and dystrophic processes with spheroids, a reduced numerical density, and a significant decrease in the area of surveillance (“microglial domain”). Consequently, there was a substantial decline in the area covered by microglia which may compromise immune protection and, therefore, neuronal survival. In vitro experiments demonstrated that soluble fractions (extracellular/cytosolic) from AD hippocampi were toxic for microglial cells. This toxicity was abolished by AT8 and/or AT100 immunodepletion, validating that soluble phospho-tau was the toxic agent. These results were reproduced using soluble fractions from phospho-tau-positive Thy-tau22 hippocampi. Cultured microglial cells were not viable following phagocytosis of SH-SY5Y cells expressing soluble intracellular phospho-tau. Because the phagocytic capacity of microglial cells is highly induced by apoptotic signals in the affected neurons, we postulate that accumulation of intraneuronal soluble phospho-tau might trigger microglial degeneration in the AD hippocampus. This microglial vulnerability in AD pathology provides new insights into the immunological mechanisms underlying the disease progression and highlights the need to improve or develop new animal models, as the current models do not mimic the microglial pathology observed in the hippocampus of AD patients.

**Electronic supplementary material:**

The online version of this article (doi:10.1007/s00401-016-1630-5) contains supplementary material, which is available to authorized users.

## Introduction

Alzheimer’s disease (AD) is so far an incurable, progressive degenerative brain disorder characterized by the presence of extracellular amyloid-beta (Abeta) plaques and intracellular aggregated phosphorylated tau, together with synaptic and neuronal cell loss. Neuroinflammation has been postulated to be a critical factor in the pathogenesis of AD [16, 17, 19]. The microglial activation process is characterized by remarkable morphological changes (larger cell body with shorter, thicker, and less branched processes) and up-regulation of proinflammatory and/or anti-inflammatory cytokines [13, 18, 44]. Activated microglia accumulate around Abeta plaques in both transgenic models [18, 22, 44] and AD patients [49, 50]. Although the inflammatory response could contribute to Abeta phagocytosis [11, 20], it has been associated with a neurotoxic detrimental effect mediated by the release of proinflammatory cytokines/chemokines and neurotoxins [16, 17, 19].

The inflammatory response in the AD brain is probably not exclusively detrimental or beneficial. In fact, recent human genome-wide association studies (GWAS) have identified multiple polymorphisms associated with the microglial immune response in AD [14, 24]. One such polymorphism, R47H, is located in TREM2 and is associated with an increased risk for late-onset AD [26]. TREM2 is a lipid sensor that, through its adapter molecule DAP12, supports Abeta-reactive microgliosis and Abeta clearance [63]. In the absence of TREM2, microglial activation is impaired. In the 5xFAD mouse model, TREM2 deficiency increased Abeta accumulation due to a dysfunctional microglial response and microglia was apoptotic instead of activated [63]. Thus, a deficient rather than an overactive microglial response could, indeed, be associated with AD development [54].

The microglial response has been preferentially studied in AD brain areas with relatively high Abeta content or in Abeta-rich transgenic models [16, 22, 49–51]. However, brain regions particularly relevant in AD development, such as the hippocampal formation, present low Abeta accumulation and a high number of phospho-tau-bearing neurons [4]. Thus, the microglial reaction in the hippocampus could be totally different than that previously described in neocortical areas. In the present work, we evaluated the microglial response in postmortem hippocampal human tissue pathologically diagnosed in Braak II to Braak V–VI stages. Contrary to the marked microglial activation reported in APP-based mice models and in human neocortical AD regions, we demonstrated a prominent degenerative process of the microglia in the dentate gyrus (DG) and CA3 of Braak V–VI samples, probably associated with the accumulation of soluble phospho-tau, as determined by in vitro assays. This degenerative process left most of the affected regions with no immunological coverage and could contribute to AD pathology progression.

## Materials and methods

### Human samples

Human autopsy specimens from the medial temporal lobe (hippocampal/parahippocampal regions) were obtained from the tissue bank Fundación CIEN (BT-CIEN; Centro de Investigación de Enfermedades Neurologicas; Madrid, Spain) and from the Neurological Tissue Bank of IDIBELL-Hospital of Bellvitge (Barcelona, Spain). The utilization of postmortem human samples was approved by the corresponding biobank ethics committees and by the “Comite de Etica de la Investigacion (CEI), Hospital Virgen del Rocio,” Seville, Spain. All cases were scored for Braak tau pathology. Table 1 summarizes the demographics of the human samples. Only Braak V–VI cases were clinically classified as demented (AD) patients. We considered the age-matched Braak II individuals as controls in our experiments.

**Table 1:** Human sample information

Braak stage	Gender		Age (years) (mean age ± SD)	Postmortem delay (mean hours ± SD)
	Male (%)	Female (%)		
(a) Unfixed frozen samples				
0 (*n* = 8)	62.5	37.5	49 ± 6	8 ± 5
II (*n* = 13)	61.54	38.46	78 ± 8.5	7 ± 4
III–IV (*n* = 9)	44.44	55.56	80 ± 11	6 ± 5
V–VI (*n* = 18)	38.89	61.11	79 ± 10	8 ± 4
(b) Fixed samples				
0 (*n* = 4)	50	50	53 ± 16	7 ± 5
II (*n* = 7)	28.6	71.4	78 ± 8.5	6 ± 3
III–IV (*n* = 10)	50	50	79 ± 7	10 ± 5
V–VI (*n* = 16)	43.8	56.20	78 ± 10	9 ± 5

For morphological studies, 4 % paraformaldehyde fixed samples were sectioned (30 µm thickness) on a freezing microtome. For molecular characterization, unfixed frozen samples were used. The anatomical boundaries of the hippocampal system and parahippocampal gyrus were identified by the anatomical and cytoarchitectonic features in sections stained with cresyl violet using a human stereotaxic brain atlas [30]. Areas between the bregma coordinates at 17 and 35 mm were analyzed.

### Antibodies

The following primary antibodies were used: (1) monoclonals, anti-phospho-tau AT100 (pSer212/Thr214) and AT8 (pSer202/Thr205, Thermo Fisher Scientific, New York, USA), anti-total tau (tau46, Cell Signaling Technology, Massachusetts, USA), 4G8 (anti-Abeta 17-24, Biolegend, San Diego, USA), 6E10 (anti-Abeta1-16, Covance, Princeton, USA), 82E1 (anti-Abeta-N-terminal, IBL, Hamburg, Germany), and anti-CD68 (PG-M1, Dako, Glostrup, Denmark); (2) rabbit polyclonals, anti-amyloid fibrils (OC, Merck Millipore, Darmstadt, Germany), anti-CD45 (Abcam, Cambridge, United Kingdom), anti-Iba1 (Wako Pure Chemical Industries, Osaka, Japan), and anti-P2ry12 (Sigma-Aldrich, Saint Louis, USA).

### Immunohistochemistry

Sections from control and diseased brains were assayed simultaneously using the same batches of solutions to minimize variability in immunolabeling conditions. After antigen retrieval (80 °C for 20 min in 50 mM citrate buffer, pH 6.0), endogenous peroxidase was inhibited (3 % H2O2/10 % methanol in PBS, pH 7.4 for 20 min) and non-specific staining was avoided using 5 % goat or horse serum (Sigma-Aldrich) in PBS. For single labeling light microscopy, sections were incubated with the primary antibody (24–72 h, at room temperature) followed by the corresponding biotinylated secondary antibody (1:500 dilution, 1 h at room temperature, Vector Laboratories), streptavidin-conjugated horseradish peroxidase (1:2000, 90 min, Sigma-Aldrich), and visualized with 0.05 % 3-3-diaminobenzidine tetrahydrochloride (DAB, Sigma-Aldrich) and 0.01 % hydrogen peroxide in PBS. The specificity of the immune reactions was controlled by omitting the primary antisera. For double or triple immunofluorescence labeling, sections were sequentially incubated with the indicated primary antibodies followed by the corresponding Alexa 488/568/405 secondary antibodies (1:1000 dilution, Invitrogen). AT100-immunolabeled sections were stained with 0.1 % thioflavin-S (Sigma-Aldrich) in 70º ethanol for 10 min. Sections were incubated in an autofluorescence eliminator reagent (Merck Millipore) following the manufacturer’s recommendations and examined under a confocal laser microscope (Leica SP5 II).

### Stereological analysis

The number of Iba1-immunopositive microglial cells was stereologically quantified according to the optical fractionator method [43] using an Olympus BX61 microscope equipped with the NewCAST software package (Olympus, Denmark). Cell counting was performed on every 6th section (with a distance of 180 µm) through the rostrocaudal axis of the hilar region (*n* = 5–6 sections/individual). The dentate gyrus boundaries were defined using a 2× objective. Cell number was counted using a 100×/1.35 objective. We used a counting frame of 1722 µm^2^ with step lengths of 131.23 µm. The numerical density (cells/mm^3^) was estimated as: ${\text{ND}} = {Q \mathord{\left/ {\vphantom {Q {\left( {\sum {A \times h} } \right)}}} \right. \kern-0pt} {\left( {\sum {A \times h} } \right)}}$, where ‘*Q*’ is the number of disector-counted somatic profiles, ‘$\sum A$’ is the area of the counting frame, and ‘*h*’ is the section thickness (30 µm).

### Image analysis

*Microglial loading* was defined as the percentage of area stained with anti-Iba1 (total microglia) or anti-P2ry12 (non-activated microglia) in relation to the total area analyzed. Digital images (2 sections/individual) were processed using the Visilog 6.3 image analysis system (Noesis, France). The Iba1- or P2ry12-immunopositive signal within the selected brain region was converted into 8-bit gray scale, and immunostained cells were identified by a threshold level mask. A fixed threshold level (ranging 160–180) was maintained throughout the image analysis of all sections from the same individual brain for uniformity. *Microglial spatial distribution* was determined by the grid analysis [3]. The dentate gyrus was photographed and processed with the Visilog 6.3 program. Digital images (4×, 2 sections/individual, *n* = 5–8 individuals per group) were binarized, and grid analysis was performed by placing a grid (354 × 354 μm/square) of 125,000 μm^2^ squares. The hilar region was divided into 30–50 regular squares, covering the total parenchymal space. The microglial spatial distribution was then estimated by measuring the Iba1-positive coverage area in each square of the grid. *Microglial domain* was defined as the area (µm^2^) covered by a single P2ry12-positive cell. This area was calculated by drawing a polygon connecting the distal end of total branches of the cell (see Fig. 3c). Microglia cells (20–30 cells per section) from Braak II and Braak V–VI sections (2 sections/individual, *n* = 5–7 individuals) were randomly selected using stereology-based sampling (NewCAST software from Olympus) and imaged under a 40× objective. The optical disector was set at 7118.3 µm^2^ with step lengths of 238.64 µm. Only microglial cells with a visible cell body were photographed. Finally, high-resolution images were processed using the Visilog 6.3 program, and the P2ry12-positive cell domain area was measured.

### Transgenic animals

Animal experiments were performed in accordance with the Spanish and the European Union regulations (RD53/2013 and 2010/63/UE) with the approval of the Committees of Animal Research from the Universities of Seville (Spain) and Malaga (Spain). APP/PS1 and Thy-tau22 transgenic animals were used. The APP/PS1mice [22, 58] over-expressed the human mutant PS1M146L and human APP751 carrying the Swedish (KM670/671NL) and London (V717I) mutations. The Thy-tau22 mice expressed human 4-repeat tau with G272 V and P301S mutations [48]. Non-transgenic mice (WT) of the same genetic background (C57/BL6) were used as controls. In this work, we used 2-, 6-, 9- and 12-month-old APP/PS1 mice; 2-, 9-, 12- and 16-month-old Thy-tau22; and age-matched WT mice (*n* = 5/age and genotype). Mice were killed (sodium pentobarbital, 60 mg/kg) and processed as described [22, 58]. Fixed brains were serially sectioned (40 µm coronal sections) and assayed for immunohistochemistry. Microglial (Iba1-positive) loading and microglial domain were calculated as described above (7–9 sections/mouse; *n* = 4/age/genotype).

### Preparation of soluble S1 fractions and sequential protein extraction

Soluble S1 fractions from either mouse models or human samples were prepared as described [23]. Briefly, human or mouse tissue was homogenized (Dounce homogenizer) in TBS (20 mM Tris–HCl, 140 mM NaCl, pH 7.5) containing protease and phosphatase inhibitors (Roche). Homogenates were ultracentrifuged (4 °C for 60 min) at 100,000×*g* (Optima MAX Preparative Ultracentrifuge, Beckman Coulter). Supernatants, S1 fractions, were aliquoted and stored at −80 °C. The pellets (P1) were extracted in RIPA buffer (1 % CHAPS, 1 % Na-deoxycholate, 0.2 % SDS, 140 mM NaCl, 10 mM Tris–HCl, pH 7.4); ultracentrifuged and supernatants, S2 fractions (intracellular particulate proteins), were aliquoted and stored. Pellets (P2) were re-extracted in buffered-SDS (2 % SDS in 20 mM Tris–HCl, pH 7.4, 140 mM NaCl), centrifuged as above and supernatants, S3 (SDS releasable proteins) were stored. Finally, the remaining pellets were extracted in SDS-urea (20 mM Tris–HCl, pH 7.4, 4 % SDS and 8 M urea).

### Sarkosyl-insoluble fraction isolation

Sarkosyl-insoluble tau was isolated as described [29]. Human hippocampi were homogenized in “homogenization buffer” (10 mM Tris, 0.8 M NaCl, 1 mM EGTA, 10 % sucrose, pH 7.4, plus protease, and phosphatase inhibitors). After centrifugation (5,000×*g*, 4 °C, 15 min), supernatants were incubated (2.5 h at 37 °C in agitation) with 1 % Sarkosyl (Sigma-Aldrich) and 1 % beta-mercaptoethanol (Sigma-Aldrich) in “homogenization buffer.” Samples were then ultracentrifuged (100,000×*g*, 4 °C, 30 min). Supernatants constituted the Sarkosyl soluble fractions, whereas pellets were the Sarkosyl-insoluble fraction. The insoluble fraction was rinsed twice and resuspended in TBS.

### Abeta and Tau quantification by sandwich ELISA

Total soluble Abeta or tau in S1 fractions was determined using a commercial sandwich ELISA (human Abeta x-40, Invitrogen; Abeta x-42, DRG; human Tau, Invitrogen) following the manufacturer’s recommendations. For each assay, 25 μg of protein from the pooled-soluble fractions were used. The ELISA experiments were repeated four times in independent experiments using triplicate replicas.

### Total RNA and protein extraction

Total RNA and proteins were extracted using TriPure Isolation Reagent (Roche) [22]. RNA integrity (RIN) was determined by RNA Nano 6000 (Agilent). Although no differences between Braak groups were observed, the RIN was lower in human samples compared with transgenic models (RIN: 4.95 ± 1.4 or 8.5 ± 0.5 for human and mouse samples, respectively). RNA was quantified using NanoDrop 2000 spectrophotometer (Thermo Fischer). Proteins were quantified using Lowry’s method.

### Retrotranscription and quantitative real-time RT-PCR

Retrotranscription (RT) (4 μg of total RNA) was performed with the High-Capacity cDNA Archive Kit (Applied Biosystems). For real-time qPCR, 40 ng of cDNA were mixed with 2× Taqman Universal Master Mix (Applied Biosystems) and 20× Taqman Gene Expression assay probes (Applied Biosystems, supplemental Table 1). Quantitative PCR reactions (qPCR) were done using an ABI Prism 7900HT (Applied Biosystems). The cDNA levels were determined using GAPDH and beta-actin. We observed a highly significant linear correlation between the cycle threshold (Ct) of both genes (beta-actin vs GAPDH, *r* = 0.912, *F*(1,46) = 157.73, *p* < 0.0001). Thus, normalization using either beta-actin or GAPDH produced identical results. Routinely, we used GAPDH as housekeeper. Results were expressed using the comparative double-delta Ct method (2-ΔΔCt). ΔCt values represent GAPDH normalized expression levels. ΔΔCt was calculated using Braak 0 for human samples or 9-month-old WT mice for transgenic models.

### Western blots

Western blots were performed as previously described [22, 23]. Abeta peptides were detected after 16 % SDS-Tris-Tricine-PAGE using PVDF membranes (Immobilon-P Transfer Membrane, Millipore) and a mixture of 6E10 (1/6000) and 82E1 (1/5000) antibodies. For Tau (Tau46, AT8 or AT100, 1/1000), proteins were loaded on 4–20 % SDS-Tris–Glycine-PAGE (Bio-Rad) and transferred to nitrocellulose (Optitran, GE Healthcare Life Sciences). Dot blots using OC were performed as previously described [22].

### Cell cultures

BV2 microglial cells were grown (37 °C and 5 % CO2) in RPMI 1640, 2 mM glutamine, 10 % (v/v) fetal bovine serum, plus penicillin/streptomycin (all from Biowest). SH-SY5Y neuronal cells (SH-control cells) were grown in DMEM-F12, 2 mM glutamine, 10 % fetal bovine serum, penicillin/streptomycin, and 1 % non-essential amino acids (Biowest). SH-SY5Y-Tau (SH-tau cells, see [8]) stably transfected neuronal cells (expressing 3R human tau) were selected using G418 (0.2 µg/ml, Biowest). For co-culture experiments, BV2 cells were plated (15.000 cells/cm^2^, 12 h) and SH-SY5Y cells were added at a ratio of 1/2.5 (SH-SY5Y/BV2 cells). Cells were co-cultured for 12 h in RPMI 1640. After co-culture, the cells were detached by trypsin and analyzed by flow cytometry. Apoptosis of SH-SY5Y cells (either control or tau-expressing cells) was induced using 1 µg/ml staurosporine B (Sigma-Aldrich) for 2 h [25]. This short treatment produced 72 ± 5.7 % of apoptotic SH-SY5Y cells. The absence of any direct effect of staurosporine B on BV2 cells was analyzed by omitting the SH-cells. Primary murine microglial or astroglial cultures were performed as previously described [22].

### BV2 treatment with S1 soluble fractions

BV2 cells (15.000 cells/cm^2^) were serum deprived for 12 h and then treated (0.1 µg soluble protein/µl of culture medium) for 12 h with soluble S1 fractions (from Braak 0 to V–VI or transgenic mice models). The effect on the cell survival was assessed by flow cytometry.

### Flow cytometry analysis of cell viability and apoptosis

The viability and apoptosis of BV2 cells were assessed using the apoptosis detection kit Annexin V-FITC (Immunostep) following the manufacturer’s specifications and were analyzed using a FACSCanto II flow cytometer (BD Services, San Jose, CA, USA). The pro-apoptotic effect of SH-SY5Y cells on BV2 cultures was evaluated by double staining the cells with CD45-PE (BV2) and Annexin V-FITC (apoptosis). Control experiments using single cell cultures (either viable or apoptotic BV2 or SH-SY5Y) were conducted to determine the discrimination settings (CD45-positive (BV2) and -negative (SH-SY5Y) cells). With this protocol, 86 ± 2.3 % of viable BV2 cells were discriminated from SH-SY5Y cells. Control experiments also demonstrated that a fraction (16 ± 4.3 %) of apoptotic BV2 cells displayed a reduction in the expression of CD45 and cannot be distinguished from SH-SY5Y cells.

### BV2 and SH-SY5Y phagocytosis assay (TAMRA assay)

Phagocytosis was assessed by loading SH-SY5Y or SH-SY5YTau cells with 5 µM TAMRA-ester (5-(and-6)-carboxytetramethylrhodamine succinimidyl ester, Sigma-Aldrich) [12]. TAMRA-loaded SH-cells were then treated (2 h) with staurosporine (1 µg/ml of culture medium; apoptotic cells) or PBS (control cells) and co-cultured with BV2 microglia (1/2.5 SH- vs BV2). Phagocytic BV2 cells were identified by CD45-PE and TAMRA fluorescence. For the inhibition of phagocytosis, cytochalasin B (5 μg/ml) was added to culture 30 min prior the experiment and maintained throughout the assay.

### Statistical analysis

Normality of data was assessed using the Kolmogorov–Smirnov test. Normally distributed data were expressed as the mean ± SD. Non-normally distributed data were represented using box-plots (Sigmaplot) or scatter-plots with the median and interquartile range (GraphPad). For normally distributed data, mean values were compared using ANOVA followed by Tukey’s test (more than two groups) or two-tailed *t* test (for two group comparisons). The data which were not normally distributed were compared by the Mann–Whitney U test (for two groups comparisons) or Kruskal–Wallis tests (more than two groups) followed by Dunn’s post-hoc test. The significance was set at 95 % of confidence. Linear correlations were analyzed using the Kendall tau-beta test. In all the cases, IBM SPSS (v23) Statistics software was used.

## Results

### Weak activation and marked degeneration of microglial cells in the AD hippocampus

**Fig. 1:**
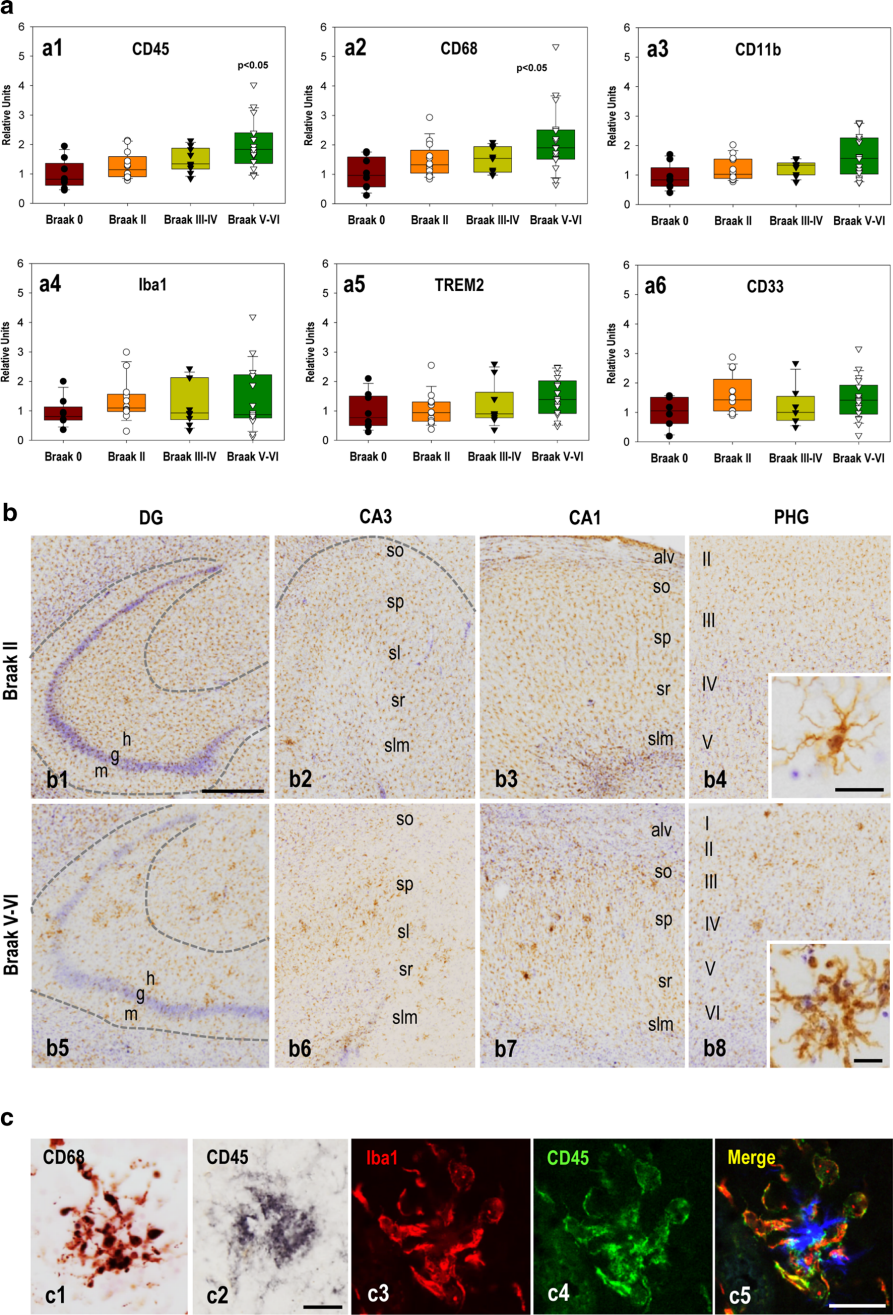
Attenuated microglial activation in the hippocampus of AD brains (Braak V–VI). **a** Microglial activation was analyzed (qPCR) in Braak 0 (*n* = 8) and age-matched Braak II (*n* = 13), Braak III–IV (*n* = 9) and Braak V–VI (*n* = 17) cases. Expression levels, normalized using GAPDH, were referenced to Braak 0. The data are represented individually (*dots*) or as *box-plots*. Significance was analyzed by Kruskal–Wallis and Dunn’s multiple comparison tests. **b** Iba1-immunostained sections of Braak II (*b1–4*) and Braak V–VI (*b5–8*) tissue. Morphological evaluation showed microglial pathology in Braak V–VI individuals (*b5–6*). **c** High magnification images of Braak V–VI microglial cells immunostained for CD68 (*c1*) and CD45 (*c2*). Activated microglia were predominantly observed forming clusters of cells surrounding Abeta plaques (*blue in c5*) as demonstrated by triple (Iba1 in *red*/CD45 in *green*/Abeta in *blue*) immunofluorescence and confocal microscopy (*c3–5*). *Alv* alveus, *g* granular layer, *h* hilus, *m* molecular layer, *so* stratum oriens, *slm* stratum lacunosum-moleculare; *sp* stratum pyramidale, *sr* stratum radiatum. *I–VI* cortical layers. *Scale bars* b1–8, 500 µm (insets in b4 and b8, 20 µm); c1–2, 25 µm; c3–5, 10 µm

We first evaluated the activation status of microglial cells from human samples by qPCR and surprisingly, markers as CD11b, Iba1, TREM2 and CD33 were not significantly altered in AD patients (Fig. 1a, a3–6). Only the expression of CD45 and CD68 was significantly increased in the Braak V-VI samples (Fig. 1a, a1–2). In addition, a significant increase in the expression of C/EPB-alpha and Pu.1 genes was also found (supplemental Fig. 1a, a1-2). Moreover, we observed significant linear correlations between the expression of all four markers (CD45, CD68, Pu.1 and C/EPBa), implying the existence of a unique cell population (supplemental Fig. 1a, a3-6). Microglial activation was also tested by immunohistochemistry using Iba1 (Fig. 1b). Morphologically, Iba1-positive cells in Braak II tissue displayed a highly ramified profile, consistent with a non-activated phenotype (Fig. 1b, b1–4). The presence of activated microglia (enlarged cell body and thickened and retracted branches) was very scarce in Braak III-IV tissue (not shown) and slightly more abundant in Braak V–VI tissue (Fig. 1b, b5–8). These cells were exclusively observed surrounding Abeta plaques, as also assessed using CD45 and CD68 markers (Fig. 1c). This limited microglial activation was in agreement with the low and late presence of extracellular Abeta deposits in the hippocampus (supplemental Fig. 1c) [4], while substantial phospho-tau accumulation was broadly distributed in the hippocampal formation in the Braak V–VI stage tissue (supplemental Fig. 1b). On the other hand, in Braak V–VI samples, an apparent low number of Iba1-positive microglial cells was observed (compare Fig. 1b, b1–2 and b5–6).

**Fig. 2:**
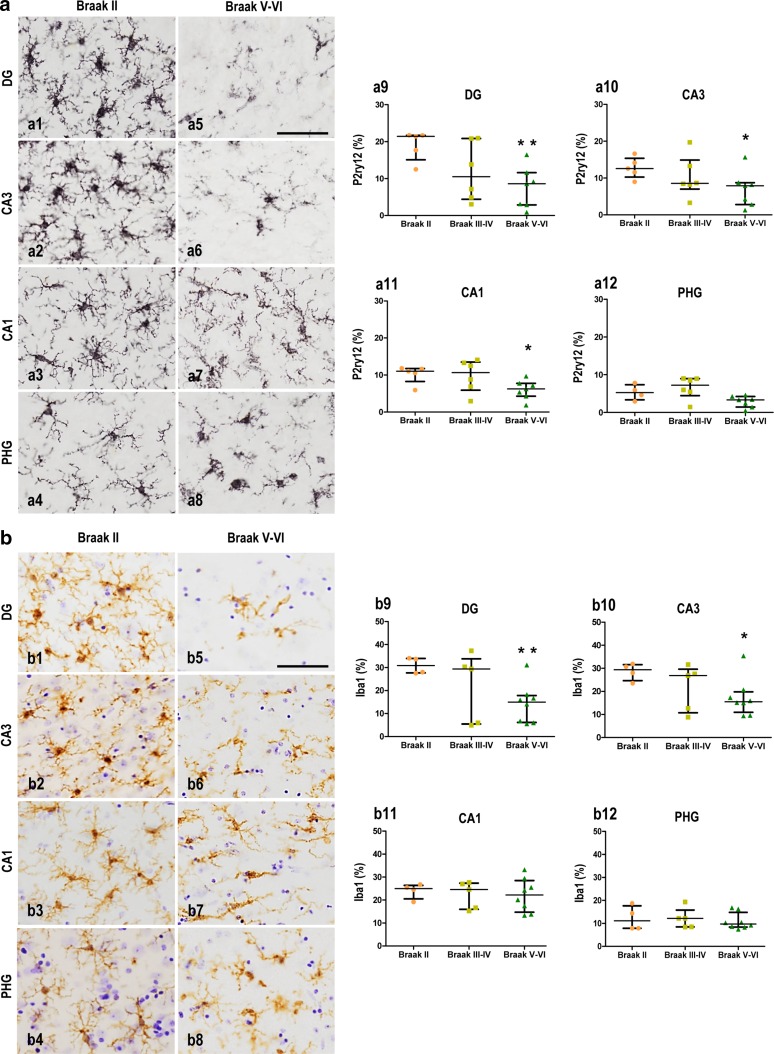
Reduced microglial load in Braak V–VI hippocampus. Microglial pathology was assessed using P2ry12 receptor (**a**) and Iba1 (**b**) immunostaining. **a** Representative images of Braak II (*a1–4*) and Braak V–VI (*a5–8*) samples in different hippocampal areas (indicated in the figure). Quantitative analysis (*a9–12*) of the area of parenchymal space (percentage) occupied by P2ry12-positive microglial cells in Braak II (*n* = 5; *orange circles*), Braak III–IV (*n* = 6; *light-green squares*), and Braak V–VI (*n* = 7; *dark green triangles*) samples. The results are shown individually (*dots*) with the median and the interquartile range. **b** Representative images from different hippocampal areas of Braak II (*b1–4*) and Braak V–VI (*b5–8*) samples immunostained for Iba1. Quantitative analysis (*b9–12*) of Iba1 load (percentage) determined in the same population as in panels *a9–12*. *p = 0.03; **p = 0.005, Mann–Whitney *U* test comparison between Braak II and Braak V–VI groups. *Scale bars* a1–8 and b1–8, 50 µm

**Fig. 3:**
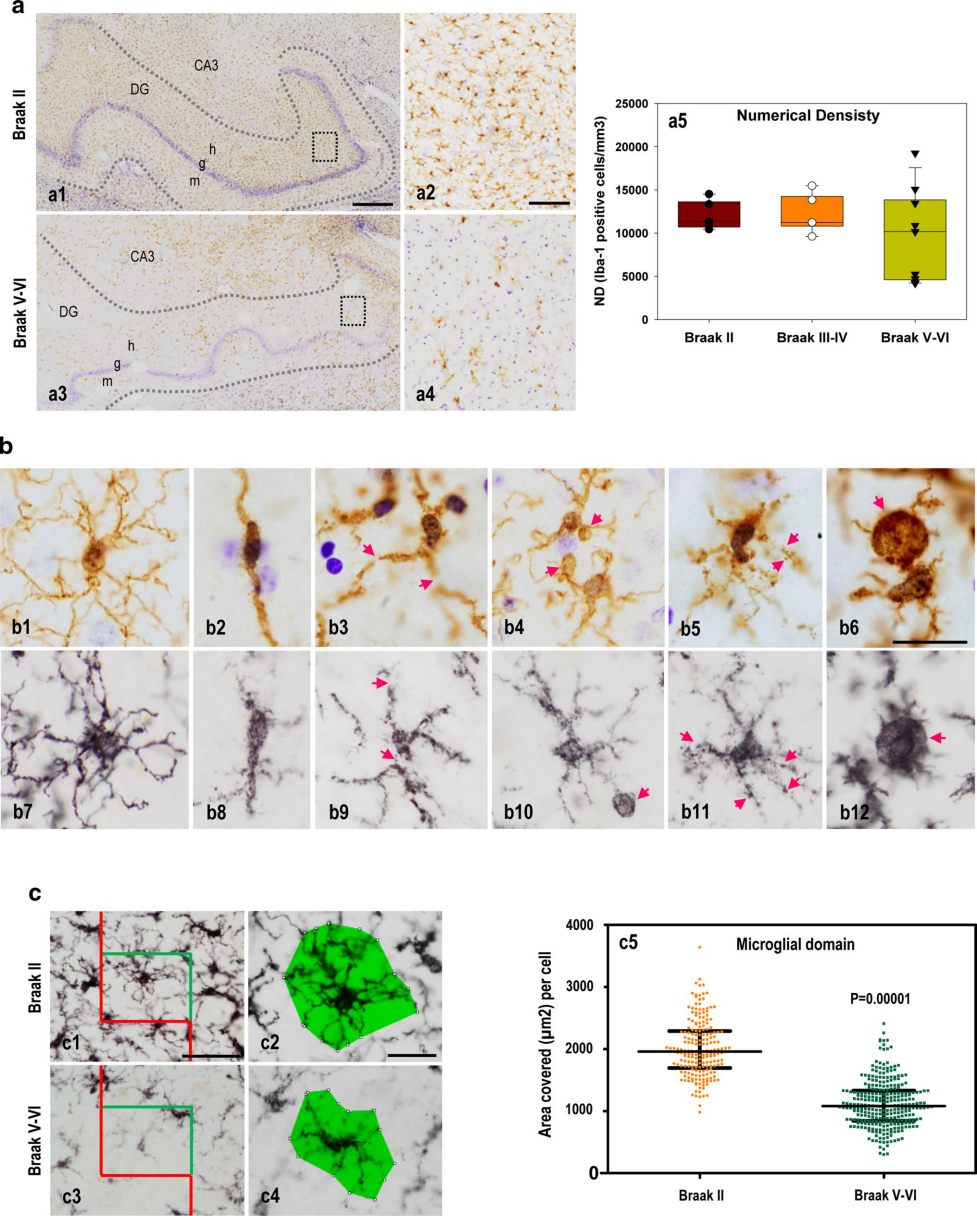
Microglial degeneration at the dentate gyrus of the Braak V–VI hippocampus. **a** Numerical density (cells/mm^3^) of Iba1-positive microglia at the hilar region from Braak II (*a1*, *boxed area a2*; *n* = 5), Braak III–IV (not shown; *n* = 5), and Braak V–VI (*a3*, *boxed area a4*; *n* = 9) cases was determined by stereology. Sections were immunostained with anti-Iba1 and counterstained with cresyl violet. The data (*a5*) are shown individually or by *box-plot*. **b** Representative high magnification images of Iba1 (*b1–6*) and P2ry12 (*b7–12*) immunostained microglial cells from Braak II (*b1* and *b7*) and Braak V–VI cases with (*b2–3* and *b8–9*) or without (*b4–6* and *b10–12*) differences in the numerical density. Abnormal morphological features of microglial cells were visualized with both Iba1 (*b1–6*) and P2ry12 (*b7–12*) immunostaining and included deramification (*b2* and *b8*), fragmentation (*b3* and *b9*), beading with spheroidal swellings (*b4–5* and *b10–11*), shortening (*b5–6* and *b10–11*), and dystrophies (*b6* and *b12*) of the processes. **c** Microglial domain (area of surveillance of a single microglial cell; *c2* and *c4*) was quantitatively determined (*c5*) in the dentate gyrus of Braak II (*n* = 5) and Braak V–VI (*n* = 8) cases. P2ry12-positive cells were randomly selected using stereology-based sampling (*n* = 200 or 300 cells from Braak II or Braak V–VI cases). Quantitative data (*dot plots* and *median plus* interquartile range) demonstrated the existence of a highly significant decrease (Mann–Whitney *U* test, *p* = 0.00001) in the microglial domain in Braak V–VI samples, compared with age-matched Braak II cases. *DG* dentate gyrus, *g* granular layer, *h*, hilus, *m* molecular layer. *Scale bars* a1 and a3, 500 µm; a2 and a4, 100 µm; b1–b12, 20 µm; c1 and c3, 50 µm; c2 and c4, 20 µm

We next assessed the percentage of area occupied by the microglial cells immunostained for the P2ry12 receptor (P2ry12 loading). As shown here (Fig. 2a, a1–a4), P2ry12-positive microglia were abundant in Braak II samples, displaying a highly ramified morphology. Significant regional differences with dentate gyrus > CA3 > CA1 > parahippocampal gyrus (PHG) were observed (Kruskal–Wallis, *p* = 0.002, *p* < 0.05 Dunn’s post-hoc test) (Fig. 2a). However, microglial cells exhibited a remarkable pathology in Braak V–VI samples (Fig. 2a, a5–a8). At the dentate gyrus and CA3 (Fig. 2a, a5–6), microglia were notably scarce and displayed clear morphological abnormalities (see Fig. 3b for details), whereas those in the CA1 and PHG appeared less affected (Fig. 2a, a7–8). This observation was quantitatively confirmed (Fig. 2a, a9–12). The quantitative analysis of the Braak III–IV samples (images not shown) showed a highly heterogeneous pattern.

The reduction in P2ry12-microglial loading could actually reflect a decrease in the total microglial population in AD samples or a down-regulation of the P2ry12 receptor following microglial activation (see supplemental Fig. 2a and also Ref. [5]). Thus, we next evaluated the area occupied by Iba1-positive microglial cells. As shown in Fig. 2b, we observed a similar pattern as above. In Braak II samples, highly ramified microglia were abundant with loads higher in the DG and CA3 than in the CA1 or PHG (Fig. 2b, b1–4; Kruskal–Wallis *p* = 0.009, *p* < 0.05 Dunn’s post-hoc test). Furthermore, Braak V–VI samples also showed a reduction in Iba1-positive microglial cell number and/or arborization (Fig. 2b, b5–8). Indeed, this observation was quantitatively corroborated (Fig. 2b, b9–12). Also in agreement with our previous data, Braak III–IV samples (not shown) displayed an intermediate pathology between Braak II and Braak V–VI samples. Thus, both microglial markers, P2ry12 and Iba1, gave similar results. In addition, we also observed a highly significant linear correlation between both P2ry12 and Iba1 levels in both the DG and CA3 (Kendall *τ* correlation analysis; *τ* = 0.50, *p* = 0.0001).

### Microglial pathology in the AD hippocampus includes reduced cell number, dystrophic morphology, reduced area of surveillance of individual cell, and altered spatial distribution

We next focused on the hilar region of the dentate gyrus, as it was the most affected hippocampal region, and quantified the numerical density (cells/mm^3^) of Iba1-positive cells using stereology (Fig. 3a). The data demonstrated no differences between the Braak II and Braak III–IV cohorts with relative constant numerical density among individuals (Fig. 3a, a5). However, Braak V–VI cases displayed two different populations. Four of the nine cases presented a clear decrease in the Iba1-positive microglial number (Figs. 3a, a3–5), whereas the other five individuals exhibited similar numbers to those in the Braak II cases, making the difference statistically non-significant. However, we did observe clear morphological differences between Braak II and Braak V–VI stages in all analyzed cases (Fig. 3b). Compared with Braak II (Fig. 3b, b1 and b7), Braak V–VI cases with (1) a low number of microglial cells (Fig. 3b, b2–3 or b8–9 for Iba1 and P2ry12, respectively), (2) an intermediate number of microglial cells (Fig. 3b, b4 and b10 for Iba1 or P2ry12, respectively), or (3) no reduction in microglial cells (Fig. 3b, b5–6 or b11–12 for Iba1 and P2ry12, respectively) exhibited an abnormal morphology, even though the extent and severity of the microglial pathology varied among the individuals. The pathology was characterized by shortened and less branched processes that usually were deformed, displaying cytoplasmic abnormalities (including spheroids) and even fragmentation (cytorrhexis), although this could be due to pathological redistribution of the microglial markers (see also Refs. [54, 57]).

The observed microglial pathology could also be reflected by a reduction in the microglial domain, the area of surveillance of an individual microglia. To quantitatively address this possibility, we measured the area covered by individual P2ry12-positive microglial cells selected by an unbiased stereological approach (see methods). We used the anti-P2ry12 antibody, because it produced better immunostaining of the thin and small microglial processes. As mentioned above, the expression of this purinergic receptor also decreased upon activation. Thus, only areas distant from plaques were included in the study. As shown in (Fig. 3c, c5), there was a severe decrease in the microglial domain in Braak V–VI compared with Braak II individuals. In fact, the microglial domain was drastically reduced from 2012.12 ± 453.44 μm^2^ per cell (*n* = 200 cells from 5 Braak II individuals) to 1119.39 ± 376.86 μm^2^ per cell (*n* = 300 cells from 7 Braak V–VI individuals; Mann–Whitney *U* test, *p* = 0.0001).

**Fig. 4:**
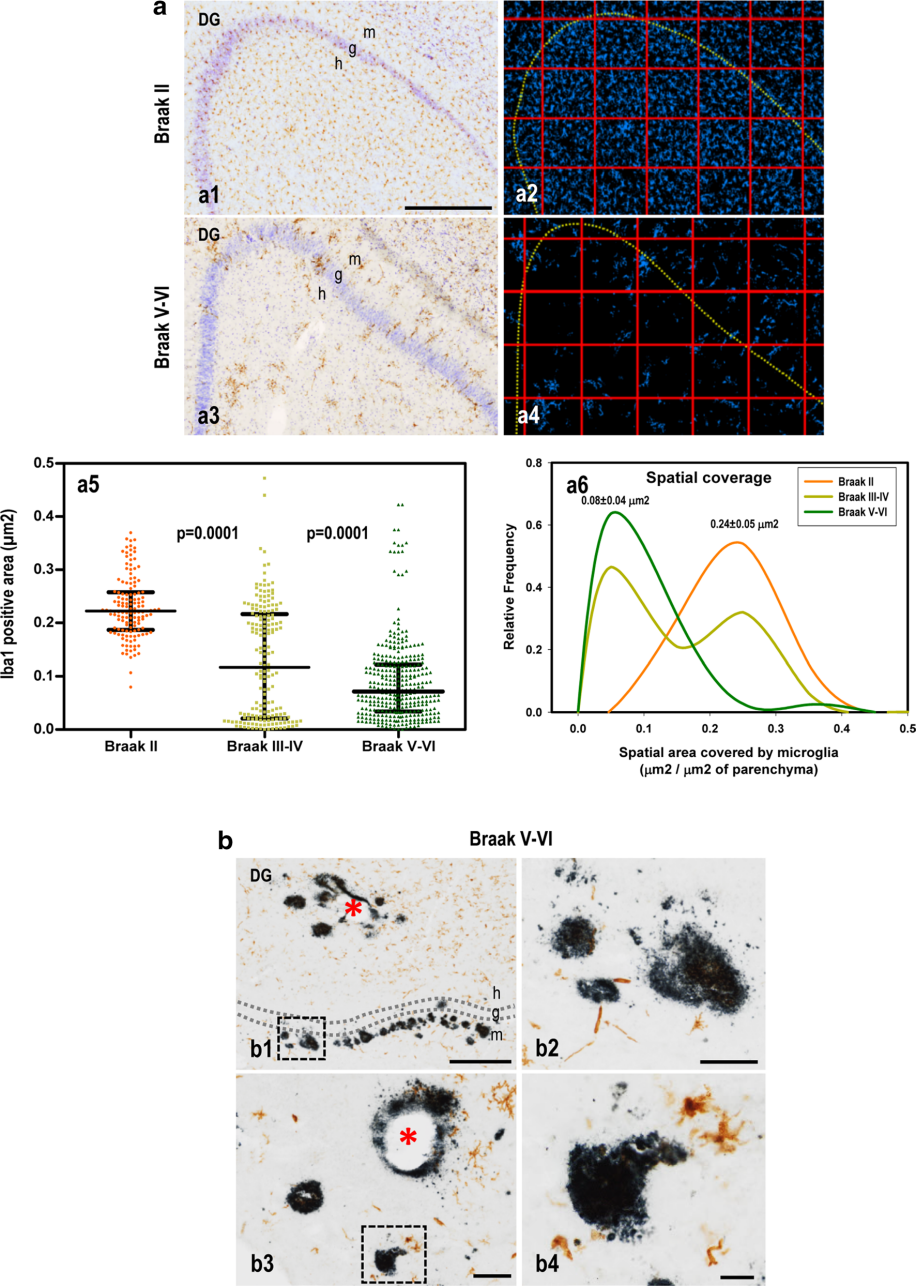
Significant reduction in the parenchymal area covered by microglial cells in the Braak V–VI dentate gyrus. **a** Microglia were immunostained using anti-Iba1, and cells were counterstained with cresyl violet (*a1*, *a3*, representative Braak II and Braak V–VI cases). The binarized images were overlaid with a grid of 30–50 regular squares (354 × 354 mm/square; *a2* and *a4*) covering the total hilar area of the dentate gyrus. The space covered (μm^2^) by microglial cells (Iba1-positive) was determined in each square (40–60 squares per sample; Braak II *n* = 4; Braak III–IV *n* = 5; Braak V–VI *n* = 9) and is individually represented in *a5* (*n* = 137, 182 or 320 individual squares for Braak II, III–IV, and V–VI, respectively). The data were analyzed by Kruskal–Wallis and Dunn’s multiple comparison tests. Significance difference between groups is indicated in the figure. (*a6*) Relative frequency distribution of each individual was calculated using IBM SPSS, and the average (±SD) is shown in the figure. **b** Representative Braak V–VI cases showing Abeta and either vascular (*b1* and *b3*) or parenchymal deposits (*b2* and *b4*) lacking microglial coverage. The sections were double immunostained using 4G8 (*dark blue*) and Iba1 (*brown*) antibodies. *DG* dentate gyrus, *g* granular layer, *h* hilus, *m* molecular layer. *Scale bars* a1, a3 and b1, 500 µm; b2 and b3, 100 µm; b4, 25 µm

The partial decrease in the microglial counts (cells/mm^3^), the abnormal morphology of the cells, and the drastic reduction in the microglial domain clearly demonstrated the existence of prominent microglial pathology in the dentate gyrus of Braak V–VI samples. Therefore, this extensive microglial pathology could also produce a reduction in the parenchymal space protected by these immune cells. To assess this possibility, we estimated the spatial distribution of microglial cells in the DG. As shown in Fig. 4a (a1–2 and a5–6), microglial cells from Braak II samples showed a regular spatial distribution, homogeneously covering the DG. This regular organization was reflected by a normal distribution of the area (Iba1-positive area) covered by the microglia (Fig. 4a, a6). These data agree well with those previously described (see Figs. 1, 2, 3) and indicate that microglial cells from aged Braak II patients were resting/non-activated. However, the same analysis in Braak V–VI samples demonstrated the existence of a clear reduction in the parenchymal area covered by the microglial cells in the hilus of the DG (Fig. 4a, a3–4 and a5–6). This reduction was not due to the migration and concentration of microglial cells around Abeta plaques, with the consequent depletion from adjacent areas, as demonstrated by the clear shift in the distribution curve to a lower covered area (Fig. 4a, a6). In fact, the area covered by microglial cells was reduced from 0.24 ± 0.06 μm^2^/μm^2^ of parenchymal space (*n* = 4) to 0.08 ± 0.04 μm^2^/μm^2^ of parenchymal space (*n* = 9; Mann–Whitney U test *p* = 0.012) in Braak II and Braak V–VI samples, respectively. Notably, in the most severely affected Braak V–VI hippocampi, microglial cells were no longer even associated with the neuritic plaques (Fig. 4b, b2 and b4) or with the vascular amyloid (Fig. 4b, b1–3), highlighting the extent of the microglial alterations.

The high heterogeneity of the microglial pathology observed in Braak III–IV individuals is also noteworthy (see Fig. 4a, a5–6). Compared with the Braak II population, three cases (from a total of five analyzed) displayed a significant decrease (Kruskal–Wallis p = 0.0001, *p* < 0.05 Dunn’s post-hoc test) in the area covered by microglial cells, whereas the other two cases were identical to the Braak II group. Although the number of cases studied was limited, the heterogeneous microglial pathology detected in these experiments was also consistent with the P2ry12 and Iba1 loads (see Fig. 2).

### The microglial degeneration of AD patients is not reproduced by transgenic mice

The microglial pathology in the Braak V–VI samples was in clear contrast to the intense microglial response in most APP-based transgenic models [16, 22]. Thus, we directly compared the microglial response in the hippocampus of our APP/PS1 model. As expected [22], we observed a massive accumulation of Abeta plaques and Abeta peptides (supplemental Fig. 3a, a1–2). This accumulation was associated with a strong microglial reaction (supplemental Fig. 3a, a3–6) characterized by a patent increase in the expression of most tested microglial genes (supplemental Fig. 3a, a7). The active microglial cells were mainly located surrounding the Abeta plaques (supplemental Fig. 3a, a3-6). A marked microglial activation was observed in the hilus of the dentate gyrus (supplemental Fig. 3a, a3, a5) along with a significant increase in the Iba1-positive microglial loading even at relatively early ages (6–9-month-old APP/PS1) (supplemental Fig. 3a, a8).

As mentioned above (supplemental Fig. 1b-c), the tau pathology in the human AD hippocampi was significantly more prominent than the Abeta accumulation. Thus, we also tested the microglial response in the Thy-tau22 model [48] (supplemental Fig. 3b). Thy-tau22 hippocampi accumulated phosphorylated tau protein principally in the CA1 pyramidal cells (supplemental Fig. 3b, b1-2). Similar to human samples, microglial cells in this transgenic model displayed a limited activation (supplemental Fig. 3b, b3-5). Only the expression of CD45, CD68, and TREM2 seemed to be slightly elevated in these mice (compare supplemental Figs. 3b, b5 and 3a, a7). Furthermore, this model also presented an attenuated microglial pathology, characterized by a reduction in the microglial arborization (supplemental Fig. 3b, b3-4) with a consequent significant decrease in the microglial domain (supplemental Fig. 3b, b6). Therefore, although attenuated, the microglial pathology in this tau model could resemble the Braak V–VI cases.

In sum, our data demonstrated the existence of a local degenerative process of the microglial cells in the AD hippocampus that was not mimicked by Abeta models and only partially by tau models.

### Soluble phospho-tau induces toxicity in microglial cells in vitro

We next investigated the factor(s) involved in microglial degeneration. Of the multiple possible factors, we focused on Abeta and phospho-tau as the major toxic proteins in AD. Furthermore, we hypothesized that soluble Abeta and/or phospho-tau would be implicated in this degenerative process. Thus, hippocampal samples were fractionated into soluble (S1, comprising both extracellular and cytosolic proteins) [23]), compartmentalized (S2, including intracellular vesicular content), aggregated (S3, SDS releasable pool), and highly aggregated (P3, Urea plus SDS releasable pool) fractions (see supplemental Fig. 4 for a detailed description).

**Fig. 5:**
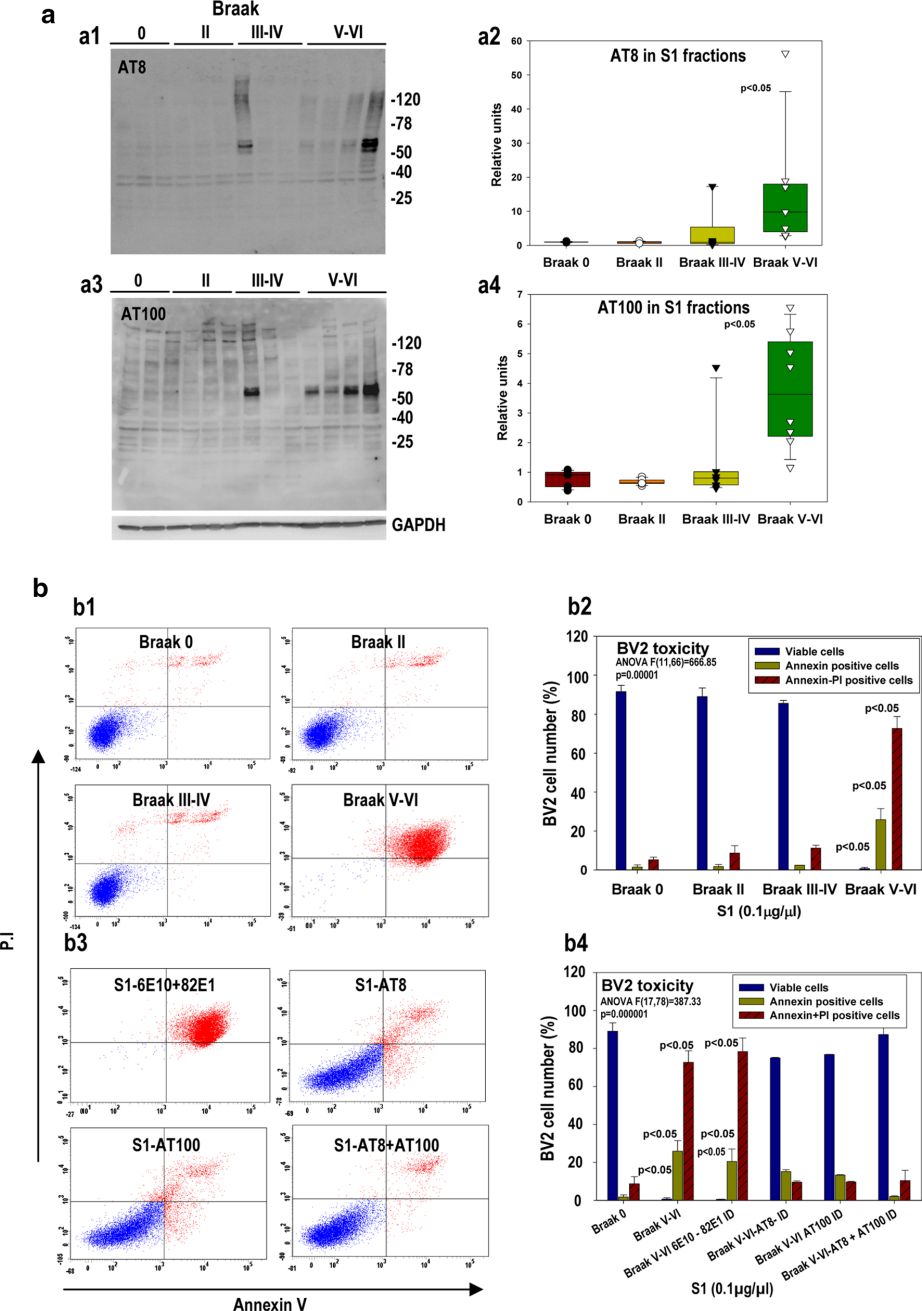
Soluble phospho-tau from the Braak V–VI hippocampus was toxic for cultured microglial cells. **a** Representative AT8 (*a1*) and AT100 (*a3*) western blots of soluble S1 fractions (extracellular/cytosolic) isolated from Braak 0 (*n* = 6), Braak II (*n* = 8), Braak III–IV (*n* = 8), or Braak V–VI (*n* = 8) individuals. GAPDH (*lower panel in a3*) was used as the loading control. The relative abundance of AT8 (*a2*) or AT100 (*a4*) phospho-tau proteins (approximately 55 kDa band) was determined by the densitometry analysis. The data were normalized by Braak 0 samples and are shown individually or as *box-plots*. Significance was determined by Kruskal–Wallis and Dunn tests. **b** BV2 microglial cells were incubated with S1 fractions from Braak 0 to V–VI samples. Toxicity was analyzed by flow cytometry. (*b1*) Representative Annexin V/propidium iodide (PI) double staining of BV2 cells treated with S1 fractions. (*b2*) Quantitative analysis of cell viability after S1 treatment. (*b3* and *b4*) Representative flow cytometry experiments and quantitative analysis of BV2 cells treated with Braak V–VI derived S1 fractions that were sham-depleted, Abeta-depleted (6E10 plus 82E1), AT8-depleted, AT100-depleted, and AT8 and AT100-depleted. In all cases, the S1 fractions were incubated with identical amounts of protein G-Sepharose. The data (*b2* and *b4*) are shown as the mean ± SD of six different experiments. Significance (indicated in the figure) was determined by ANOVA and Tukey post-hoc test

Quantitative analysis of S1 fractions (Fig. 5a) demonstrated the preferential accumulation of soluble AT8 (Fig. 5a, a1–2) and AT100 (Fig. 5a, a3–4) phospho-tau in the Braak V-VI group. With the exception of a single Braak III–IV case (from 9 cases tested), the presence of phospho-tau in the cytosolic/extracellular soluble S1 fraction was exclusively detected in AD samples. However, the total soluble tau levels were similar between the different groups (supplemental Fig. 4a, a2). The Abeta content of these samples was low, as previously reported [23].

We next evaluated, in vitro, whether these soluble S1 fractions were toxic for BV2 microglial cells. Pooled-soluble fractions (0.1 μg of soluble protein/μl of culture media) from Braak 0 (6 individuals), Braak II (10 individuals), and Braak III–IV samples (9 individuals) produced no appreciable toxicity on BV2 cells (Fig. 5b, b1–2). Approximately 90 % of BV2 cells remained viable after treatment. However, under the same experimental conditions, pooled S1 fractions from Braak V–VI samples (12 individuals) produced a strong toxic effect on BV2 cells. After 12 h of incubation, we observed a highly significant increase in early and late-apoptotic cells and only 1.0 ± 0.7 % (*n* = 6 independent experiments) of cells remained viable (Fig. 5b, b1–2). This effect was dose-dependent (not shown), and, in fact, the Braak V-VI S1 fractions presented a highly toxic potency (EC50 = 0.009 ± 0.007 μg/μl; n = 3). Soluble fractions from Braak 0, Braak II, or Braak III–IV had no effect in the range tested (from 0.1 ng to 0.1 μg of S1 protein per microliter of culture media).

To assess whether soluble Abeta or phospho-tau was the potential toxic agent, Braak V–VI derived S1 fractions were immunodepleted with either 6E10 + 82E1 (total Abeta) or AT8, AT100, and AT8 + AT100 (phospho-tau depletion) antibodies. The treatment with 6E10 + 82E1 antibodies produced absolutely no reduction on the toxicity of the S1 fractions (Fig. 5b, b3–4). However, AT8, AT100, and the combination of both antibodies produced a partial (for either AT8 or AT100 antibodies) or a complete suppression (for the combination of AT8 and AT100 antibodies) of the S1 toxicity on BV2 cells (Fig. 5b, b3–4).

It could be argued that this toxic effect was non-specific and simply reflects the toxicity of soluble proteins from Braak V–VI brain samples. To test this possibility, we performed several control experiments. First, we tested whether aggregated Sarkosyl-insoluble phospho-tau (supplemental Fig. 5a, a1) was also toxic for BV2 cells. However, this phospho-tau fraction produced absolutely no toxicity (supplemental Fig. 5a, a2-3). Second, soluble fractions prepared from APP/PS1 transgenic models, despite the relatively high oligomeric Abeta content (supplemental Fig. 6a, a1) and the strong microglial activation (supplemental Fig. 3a, see above and [22]), produced no reduction in BV2 viability (supplemental Fig. 6a, a2). Third, in accordance with the putative toxic effect of soluble phospho-tau, aged transgenic Thy1-tau22 mice accumulated soluble phospho-tau (AT8-positive) in the S1 fractions (supplemental Fig. 6b, b1), and, consequently, the S1 fractions from these mice produced a significant reduction in the number of viable BV2 cells (supplemental Fig. 6b, b2). Notably, S1 fractions isolated from younger tau transgenic animals (2 and 9 months of age) showed lower levels of AT8-positive tau and produced no effect on BV2 viability (supplementary Fig. 6b, b2). Fourth, this effect was not a general cytotoxic effect produced by soluble phospho-tau, as demonstrated by the absence of a toxic effect of Braak V-VI-derived S1 fractions on the astroglial cell line WJE (supplemental Fig. 5b, b1-2) and primary murine astroglial cultures (not shown). Finally, a similar toxic effect of S1 fractions from Braak V–VI samples was also reproduced in murine primary microglial cells (viable cells 66.10 ± 16.12; 58.69 ± 6.39 and 15.46 ± 6.64 %, for PBS, Braak II and Braak V–VI, respectively; *n* = 3 for PBS or *n* = 5 for Braak II and V–VI; ANOVA *F*(2,10) = 55.06, *p* = 0.0001; Tukey *p* < 0.05). Therefore, these data demonstrated that soluble phospho-tau, present predominantly in Braak V-VI samples, was pro-apoptotic for microglial cells in vitro.

As mentioned above, the S1 fractions contained both extracellular and cytosolic soluble proteins. Thus, although the existence of extracellular tau is now considered crucial for AD propagation [1, 9, 56], phospho-tau could be predominantly accumulated in the cytosol. Therefore, it is likely that most of the phospho-tau in our S1 fractions were originally located intraneuronally. However, it is also known that microglia cells could phagocytose apoptotic tau-bearing neurons [29, 35]. Thus, we also tested whether intracellular phospho-tau could be toxic for microglia after phagocytosis. For these experiments, we co-cultured BV2 cells with SH-SY5Y cells constitutively expressing 3R human tau (SH-tau cells). These SH-tau cells accumulated soluble intracellular phospho-tau (supplemental Fig. 6c). Non-transfected SH-SY5Y cells were used as control cells (SH-control cells).

**Fig. 6:**
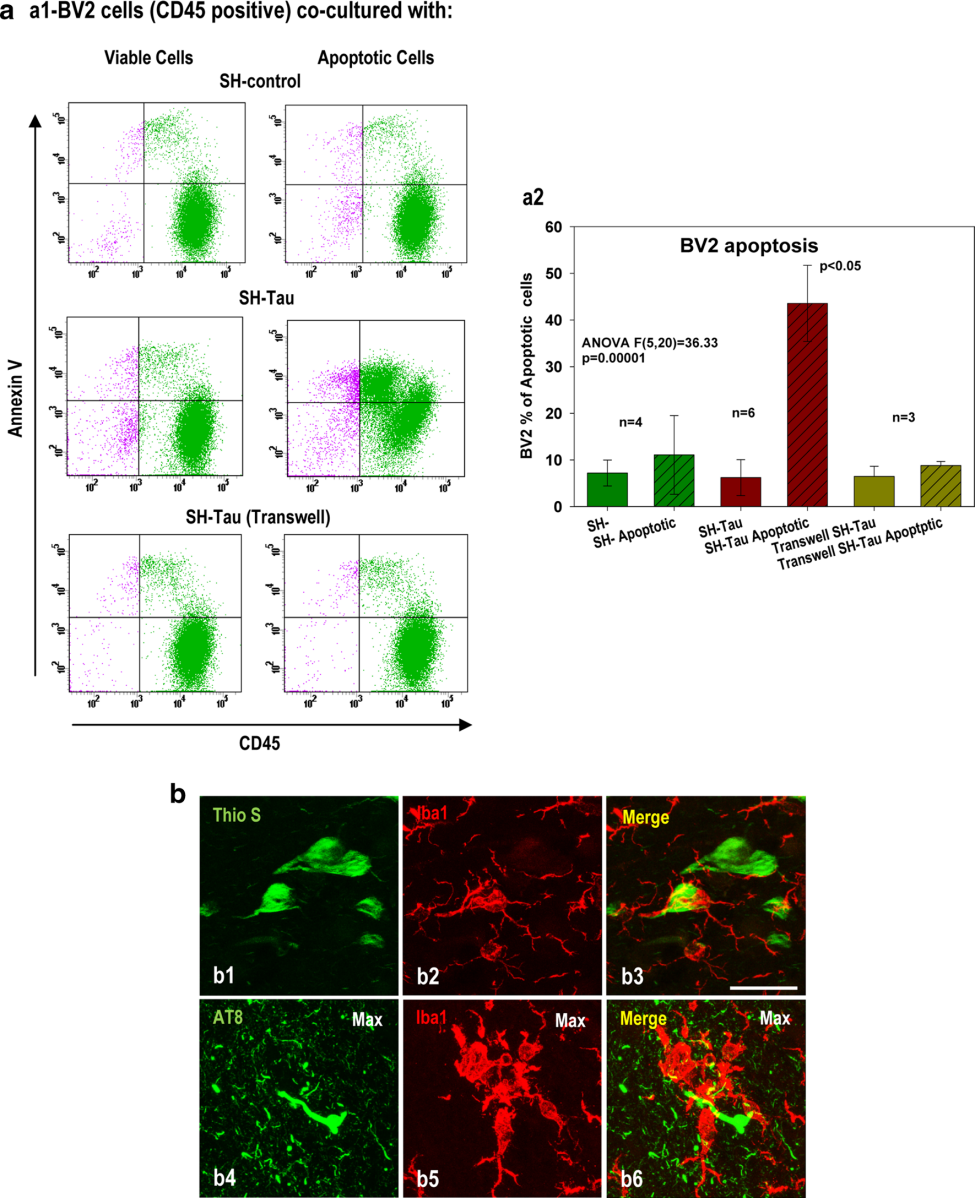
Phagocytosis of apoptotic SH-SY5Y cells expressing human tau was toxic for BV2 cells. **a** Flow cytometry analysis of BV2 apoptosis (CD45- and Annexin V-positive cells) induced after co-culture with viable or apoptotic SH-control or SH-tau cells. (*a1*) Representative double staining of CD45/Annexin V BV2 cells co-cultured with viable (*left panels*) or apoptotic (*right panels*) SH-SY5Y cells (SH-control; upper panel), SH-SY5Y cells transfected with Tau (SH-tau; *middle panel*), and SH-tau cells co-cultured into a transwell device (*lower panel*). (*a2*) Quantitative analysis of apoptotic BV2 cells (percentage) selected by CD45 and Annexin V double staining. The number of independent experiments was indicated in the figure. The data are shown as the mean ± SD. Significance (indicated in the figure) was determined by ANOVA and Tukey post-hoc test. **b** Representative Braak V–VI hippocampus images with double Iba1 and AT8 immunofluorescence and laser confocal microscopy showing microglial cells in very close contact with phospho-tau-positive neuronal somata (*b1–3*) or neuropil threads (*b4–6*) suggestive of microglial-mediated phagocytosis of affected neurons. *Scale bar* 20 µm

First, we evaluated whether BV2 microglial cells (CD45-positive cells) were able to phagocytose apoptotic SH-controls cells. In fact, control experiments using TAMRA-loaded viable or apoptotic SH-control cells demonstrated BV2 actively phagocytosed apoptotic cells (TAMRA-positive BV2 cells: 7.5 ± 3.5 vs 70.2 ± 4.66 % in presence of control and apoptotic SH-cells, respectively, *n* = 3, see also [12]). Second, we tested whether phagocytosis of SH-control cells was toxic for BV2 microglia. Our data demonstrated that phagocytosis of SH-control cells was not toxic for BV2 cells. As shown in Fig. 6a (a1–2), co-culture of viable (75.7 ± 5.1 % viable cells, *n* = 4) or apoptotic (72 ± 5.7 % apoptotic cells, *n* = 4) SH-cells produced low or non-toxicity on BV2 microglial cells. Moreover, co-culture of viable (84.7 ± 4.5 % viable cells, *n* = 6) SH-tau-cells were also non-toxic for BV2 cells (Fig. 6a, a1–2). Thus, the phagocytic process of staurosporine-induced apoptotic cells was not toxic for BV2 cells, and, furthermore, the presence of intracellular soluble phospho-tau in viable neuronal cells, such as SH-tau, was also not toxic for microglia. However, co-incubation of BV2 cells with apoptotic SH-tau cells (79.3 ± 8.4 % of apoptotic cells, *n* = 6) produced a clear toxic effect on this microglial cell line (Fig. 6a, a1–2). In fact, 43.55 ± 8.18 % (*n* = 6; ANOVA *F*(5,20) = 36.33, *p* = 0.0001, Tukey *p* < 0.05) of BV2 cells were annexin V-positive under these conditions. Therefore, phagocytosis of phospho-tau-bearing apoptotic cells seemed to be toxic for microglia.

The implication of active phagocytic activity in the toxic process was also probed by the physical inhibition of phagocytosis using a transwell device. As shown in Fig. 6a (a1–2), the inclusion of apoptotic SH-tau-cells into the transwell completely prevented BV2 toxicity. Furthermore, the pharmacological inhibition of phagocytosis, using cytochalasin B (from 79.96 ± 2.25 to 25.3 ± 1.15 % of phagocytic BV2 cells, using the TAMRA assay, *n* = 3), produced a significant inhibition in BV2 toxicity induced by apoptotic SH-tau-cells (62.63 ± 4.47 vs 36.00 ± 3.41 %, *n* = 4; in the absence or in the presence of cytochalasin B, respectively; two-tailed *t* test *t* = 9.38, *p* = 0.0001). As an additional control experiment, we also tested whether Abeta-accumulating neuronal cells, such as N2a-APPswe cells [58, 61], were toxic after phagocytosis. As expected, co-culture of viable or apoptotic N2a-APPswe cells did not produce toxicity on BV2 microglia (11.38 ± 0.34 vs 11.31 ± 3.51 %, *n* = 4, for viable or apoptotic N2a-APPswe cells, respectively). Therefore, either extracellular (through a possible direct effect) or intracellular (through a phagocytic process) phospho-tau was toxic for microglial cells, whereas soluble Abeta seemed to have a little to no effect under these conditions. This hypothesis is in agreement with the multiple microscopic images displaying microglial cells surrounding and, probably, phagocytosing neurofibrillary tangle-bearing neurons (Fig. 6b, b1–3) and tau-positive neuropil threads (Fig. 6b, b4–6) in the hippocampus of the Braak V–VI samples.

In conclusion, the release of soluble phospho-tau species or the phagocytosis of neurons containing soluble phospho-tau produced a toxic effect on microglial cells.

## Discussion

The role of the inflammatory response in Alzheimer’s disease is far from elucidated. In this study, we evaluated the microglial response in the hippocampus of postmortem human samples classified from Braak 0 to Braak VI stages. Contrary to the expected results based on APP-transgenic models [16, 22, 33], an attenuated rather than massive microglial activation was observed in Braak V–VI samples (AD cases). More importantly, the present results also demonstrate the existence of a microglial degenerative process in the Braak V–VI hippocampus. This degenerative process was more prominent in the hilar region of the dentate gyrus and the CA3 region of the hippocampus proper. The microglial pathology is characterized by the following: (1) the presence of degenerative microglial cells, which exhibit shortened and less branched processes, cytoplasmic fragmentation (cytorrhexis) and spheroids formation (see also [54, 57]); (2) a reduction (at least in 4 of 9 Braak V–VI cases tested) in the microglial numerical density; and (3) a dramatic decrease in the area of surveillance of individual microglial cells, described here as the microglial domain. These pathological modifications produce a prominent decrease in the parenchymal area covered by microglia in these particular hippocampal subfields (DG and CA3), leaving most of the parenchymal space with no immune coverage, including Abeta plaques and vascular Abeta depositions.

It could be argued that this microglial degenerative process reflects the advanced pathological status of demented Braak V–VI individuals. If this is the case, we speculate that non-demented cases with lower pathology (Abeta and/or tau accumulation), such as Braak II or Braak III–IV samples, could better reflect the early microglial response [47]. However, microglial cells from Braak II individuals presented a highly ramified morphology with a regular spatial distribution, covered the total parenchymal space, and displayed absolutely no signs of activation or degeneration. Furthermore, neither molecular nor morphological evaluation of the Braak III–IV samples indicated the existence of microglial activation outside of that restricted to Abeta plaques or vascular deposits. Concerning microglial degeneration, Braak III–IV samples were highly heterogeneous. Approximately 50 % of the tested Braak III–IV population displayed similar morphological and molecular parameters to that of the Braak II population, whereas the other 50 % better resembled the Braak V–VI cases (see Figs. 2, 4). We do not know what determines this heterogeneity. However, the existence of a highly significant correlation between two different microglial markers, P2ry12 and Iba1, in both the DG and CA3 subfields from samples of different Braak pathology (from Braak II to Braak V–VI individuals) strongly suggests that the microglial degeneration in Braak V–VI patients, at these particular hippocampal areas, could be part of a continuum along with the progression of the AD.

As mentioned above, microglial degeneration was particularly evident in the hilus of the dentate gyrus and in the CA3 subfield. Interestingly, these two hippocampal areas also displayed the highest microglial loading compared with other hippocampal subfields in Braak II individuals. Although we do not know what determines this microglial regionalization, it is obvious that the higher microglial load in Braak II samples and the microglial degeneration in AD patients seem to be associated with the DG-CA3 connection by the mossy fibers. In this sense, recent studies have suggested the existence of hippocampal hyperexcitability in both AD patients [2] and transgenic models [40, 60]. This hyperactive status is particularly evident at the level of DG to CA3 connection. Although we cannot conclude that a hyperactive status of the mossy fibers is involved in the microglial degeneration observed in AD patients based on the present data, this idea could be a potential explanation, and further investigation is clearly needed.

The implication of microglial cells in the development of neurodegenerative disorders is generally accepted [16, 19, 59]. However, microglial dysfunction in AD has been primarily associated with over-activation and cytotoxicity of these cells [19, 59]. Microglial activation has been undoubtedly observed in cerebral regions with the early and abundant extracellular Abeta deposits [15, 36, 49, 50]. In fact, microgliosis has been associated with the disease duration and the neurodegenerative progression of AD [49–51]. Furthermore, in the APP-based transgenic models, microglial activation may drive the AD pathology. In this sense, increases in microglial reactivity [32] or, in contrast, pharmacological depletion of microglial cells [32, 36, 53, 63] clearly enhanced or reduced Alzheimer’s disease pathology, respectively, with consequent changes in tau phosphorylation, synaptic strength, and neuronal cell lost. Therefore, the data presented here seem to contradict those previously reported. The most parsimonious explanation to reconcile these, apparently, contradictory observations is the different Abeta and/or tau content between different brain regions in AD patients and/or animal models. Most of these observations are based on Abeta producing mice, similar to our APP/PS1 model, or in Abeta-rich cortical areas from AD patients. However, the hippocampus of AD patients exhibits low and late Abeta pathology, whereas phospho-tau accumulates starting in the early stages of the disease [4]. Thus, the preferential accumulation of phospho-tau over Abeta plaques could induce a totally different microglial response.

The pathological consequence(s) of a deficient immunological protection due to the microglial degeneration observed in AD patients are unknown. Microglial cells are implicated in the maintenance of synaptic integrity [62] and, in fact, could promote learning-dependent synaptic formation [41]. In addition, microglia is involved in Abeta phagocytosis [27, 64, 65], senile plaque compaction, and limitation of Abeta toxicity [7, 66]. Furthermore, microglia play a relevant role in removing damaged neurons and neuronal components, such as aberrant synaptic terminals or demyelinated axons. In this sense, deficiencies in key genes for microglial survival and/or proliferation (such as CSF1R or TREM2) are associated with rare hereditary neurodegenerative diseases, such as adult-onset leukoencephalopathy with axonal spheroids or Nasu–Hakola disease (respectively) [6, 38, 39]. In both diseases, the microglial response and, more relevant, the microglial survival seem to be compromised. Moreover, the rs75932628 polymorphism results in an R47H missense mutation in TREM2 and increases the risk for late-onset AD [14, 26, 37, 65]. Furthermore, TREM2 knock-out models display dystrophic microglial cells similar to that described in this work [42], deficiencies in microglial survival, and aggravation in AD pathology [63, 65]. Although the presence of any of these rare TREM2 variants in our limited AD cohort is highly improbable, these studies together with our present data strongly suggest that microglial pathology, with the consequent deficient immunoprotection in relatively large areas of the hippocampus, such as the dentate gyrus and CA3, might, indeed, contribute to the progression of AD pathology and cognitive impairment.

Another relevant finding of our work is the demonstration of the toxic effect of soluble intra- and/or extracellular phospho-tau on microglial cells by in vitro assays, which strongly supports the hypothesis that phosphorylated tau is the putative toxic agent for microglia in the AD hippocampus. Furthermore, our in vitro experiments are also in concordance with the differential microglial response to the Abeta or phospho-tau pathology observed in transgenic models and with the neuroprotection observed in tau-deficient models [31, 45]. However, we are also aware that the microglial pathology in the tau model was modest and less obvious than what was observed in the Braak V–VI samples. We do not know why this apparent discrepancy exists, and it clearly indicates that AD pathology is more complex than what is reproduced in mouse models.

We do not know which form(s) of phospho-tau (monomeric vs oligomeric) exerts the toxic effect on microglial cells. However, it is important to emphasize that not all phospho-tau species present in AD samples are toxic for microglial cells, as demonstrated using Sarkosyl-insoluble phospho-tau. It has long been recognized that Sarkosyl-insoluble phospho-tau is part of the intracellular paired helical filaments that constitute the basis for neurofibrillary tangle formation [28]. Therefore, as proposed by others, tangle formation could represent a cellular protection mechanism that prevents neuronal toxicity [46] and, as probed in this work, microglial toxicity. The cause of the accumulation of soluble phospho-tau species in AD patients is currently unknown.

Another open question is whether soluble phospho-tau is released by the neurons or, on the contrary, is retained intracellularly. Although there is evidence that supports the neuronal release of tau [1, 9, 10, 56], our data also demonstrate that soluble intracellular phospho-tau could be toxic for microglia after phagocytosis. The phagocytic capacity of microglial cells is highly induced by apoptotic signals in the affected neurons [34, 52]. Thus, we postulate that not only the accumulation of intraneuronal soluble phospho-tau but also the induction of apoptosis and the consequent phagocytosis of the phospho-tau affected neurons by the microglia trigger the toxicity in these cells, although the exact intracellular/extracellular cell death mechanism needs to be further investigated. Supporting this suggestion, viable SH-tau cells produced no toxicity, whereas apoptotic tau-expressing SH-cells were highly toxic for BV2 cells. Moreover, phagocytosis of Abeta-expressing N2a-APPswe cells was not toxic for microglial cells. Furthermore, the toxic effect was suppressed by physical (transwell device) and pharmacological (cytochalasin B) inhibition of phagocytosis. Therefore, the toxic effect of soluble phospho-tau in AD cases is likely mediated by the microglial phagocytic capacity for the clearance of apoptotic tau-bearing neurons or, more probably, for the elimination of the neuropil threads. This suggestion is in agreement with a recent work [21] that demonstrates the involvement of microglia in the phagocytosis of synapses after oligomeric Abeta stimulation and the accumulation of soluble phospho-tau in the presynaptic terminals of demented cases [55]. Thus, based on these observations, we postulate that the accumulation of soluble phospho-tau forms in aberrant synaptic terminals and/or dystrophic neurites could induce the phagocytic response of the microglial cells, thereby producing toxicity in these cells. This close association between tau-bearing neurons or neuropil threads and severely dystrophic microglial cells has also been reported by Streit and co-workers [54].

This restricted toxic effect, together with a probably limited regenerative capacity of the microglial cells in AD individuals, could explain the regional pattern of microglial degeneration and also the minor microglial pathology observed in other hippocampal regions, such as CA1 or CA2, with a high accumulation of tangle-bearing neurons.

In summary, our results demonstrate the existence of a significant microglial degenerative process in the hippocampus of AD patients. This degenerative process reduces the parenchymal area covered by microglia and consequently compromises the immune coverage and neuronal survival. Our data also demonstrate that soluble AT8- and/or AT100-positive phospho-tau species, located either extracellular or intracellular after phagocytosis, drive microglial degeneration. The microglial vulnerability in AD pathology provides new insights into the immunological mechanisms underlying this neurodegenerative disease. Finally, our findings highlight the need to improve or develop new animal models, as the current models do not mimic the microglial pathology observed in the hippocampus of AD patients.

## Electronic supplementary material

Below is the link to the electronic supplementary material.

Supplementary material 1 (PDF 2200 kb)
