# Supplementary material for: Soluble phospho-tau from Alzheimer’s disease hippocampus drives microglial degeneration
Source: Acta Neuropathol. 2016 Oct 14;132(6):897–916. doi: 10.1007/s00401-016-1630-5 (PMC5106501; doi:10.1007/s00401-016-1630-5)
Supplement: Supplementary file 1 — Supplementary material 1 (PDF 2200 kb) [file 401_2016_1630_MOESM1_ESM.pdf]

Soluble phospho-tau from Alzheimer's disease hippocampus drives microglial degeneration

Elisabeth Sanchez-Mejias1,4*, Victoria Navarro2,3,4*, Sebastian Jimenez2,3,4, Maria Sanchez-Mico2,3,4, Raquel Sanchez-Varo1,4, Cristina Nuñez-Diaz1,4, Laura Trujillo-Estrada1,4, Jose Carlos Davila1,4, Marisa Vizuete2,3,4, Antonia Gutierrez1,4 ¶ and Javier Vitorica2,3,4¶

1-Dept. Biologia Celular, Genetica y Fisiologia. Facultad de Ciencias, Universidad de Malaga, 29071 Spain

2-Departamento Bioquímica y Biología Molecular, Facultad de Farmacia, Universidad de Sevilla, Spain.

3-Instituto de Biomedicina de Sevilla (IBiS)-Hospital Universitario Virgen del Rocío/CSIC/Universidad de Sevilla, Spain.

4-Centro de Investigación Biomédica en Red sobre Enfermedades Neurodegenerativas (CIBERNED), Madrid, Spain.

* These authors contributed equally in this work

¶ Co-Senior corresponding author

Corresponding authors

Javier Vitorica

Dept. Bioquimica y Biologia Molecular

Facultad de Farmacia. Universidad de Sevilla

Instituto de Biomedicina de Sevilla (IBiS)-Hospital Universitario Virgen del Rocío/CSIC/Universidad de Sevilla, Spain

C/ Prof. Garcia Gonzalez 2, 41012 Sevilla, Spain

Telephone: 34-95-4556770

Email: vitorica@us.es

Antonia Gutierrez

Dept. Biologia Celular, Genetica y Fisiologia.

Facultad de Ciencias, Universidad de Malaga, Spain

Telephone: 34-952133344

Email: agutierrez@uma.es

Supplemental Table 1. Human and murine Taqman probes used for qPCR

Probes	Murine	Human
c/EBPa		Hs00269972_m1
Pu.1		Hs02786711_m1
Iba1	Mm00479862_g1	Hs00610419_g1
CD11b	Mm00434455_m1	Hs00355885_m1
CD45	Mm01293577_m1	Hs04189704_m1
CD68	Mm03047343_m1	Hs02836816_g1
TREM2	Mm04209424_g1	Hs00219132_m1
CD33		Hs01076281_m1
P2rx4		Hs00602442_m1
CCR2	Mm00438270_m1	Hs00704702_s1
Cx3Cr1	Mm02620111_s1	Hs01922583_s1
P2ry12	Mm01950543_s1	Hs01881698_m1
GAPDH	Mm99999915_g1	Hs03929097_g1
Beta-actin	Mm02619580_g1	Hs99999903_m1

Supplemental Figures

Supplemental Figure 1. Microglial activation and pathological characterization of Braak II, III-IV and V-VI hippocampus.

a) The expression of microglial markers Pu.1 (a1) and c/EBPa (a2) was determined by qPCR. The expression of these two markers was increased significantly (Kruskal-Wallis and Dunn post-hoc test, see figure) only in Braak V-VI cases, as compared with Braak 0 or Braak II samples. All affected microglial markers showed significant linear correlations using tau-Kendall (a3-6), corroborating the existence of microglial activation and the implication of a single cell population. b-c) Neuropathological characterization of phospho-tau and Abeta in human hippocampal samples using immunohistochemistry with AT8 (b1-6) or 4G8 (c1-6) antibodies. Phospho-tau accumulation was clearly observed in Braak III-IV samples, mainly in CA1 field (b5) and in both CA1 (b6) and dentate gyrus (b3) in Braak V-VI stages. The Abeta plaques affected the CA1 region and molecular layer of dentate gyrus in Braak III-IV cases (c2 and c5), however the hilar region was only affected in Braak V-VI individuals (c3 and c6). Alv, alveus; g, granular layer; h, hilus; m, molecular layer; so, stratum oriens; slm, stratum lacunosum-moleculare; sp, stratum pyramidale; sr, stratum radiatum. Scale bars: b1-b6 and c1-c6, 1 mm.

Supplemental Figure 2. The expression of P2ry12 was reduced in activated microglial cells.

a) The co-localization (a3 and a6) between Iba1 (a1 and a4) and P2ry12 (a2 and a5) microglial specific markers was assessed by laser confocal microscopy in Braak V-VI samples. Co-localization (a3 and a6, arrows) between both markers was clearly observed in microglial cells not in direct contact with Abeta plaques (visualized in blue colour). The boxed regions, at higher magnification in insets, showed activated Iba1 positive and P2ry12 negative microglial cells, closely associated with Abeta plaques (in blue). Furthermore, ex vivo experiments, using isolated adult mice microglia, indicated that the P2ry12 expression was slightly reduced after LPS activation (1.05 ± 0.40 vs 0.65 ± 0.15 relative units for PBS or LPS respectively, n=4, two-tailed t-test, t=2.08,

p=0.075). Thus, the expression of P2ry12 receptor was down-regulated after microglial activation

g, granular layer. Scale bars: a1-a6, 50 µm (insets in a3 and a6, 20 µm)

Supplemental Fig. 3 Microglial response in Abeta (APP/PS1) and tau (Thy-tau22) transgenic models.

a) APP/PS1 model displayed a strong age-dependent accumulation of Abeta-plaques (a1; 12-month-old mice) and total Abeta (a2, representative western-blot using 6E10+82E1) in the hippocampal formation. This Abeta accumulation was associated with a clear microglial activation. Representative images showing Iba1 (a3, panoramic image; a4, high magnification) or CD45 (a5, panoramic image; a6, high magnification) immunostained microglial cells from 12-month-old mice. The microglial activation was also assessed by determining (qPCR) the expression (a7) of microglial markers (ANOVA and Tukey post hoc test; * $p<0.05$). Data (n=5 animals per age) were normalized using 9-month-old WT mice (n=5; not shown) and are mean ± S.D. Quantitative Iba1-load determinations at the hilar region (a8), from 6- or 12-month-old mice, also demonstrated the existence of a significant (ANOVA and Tukey post hoc test; * $p<0.05$; n=5/age and genotype) increase in the area covered by Iba-1 positive cells, as compared with age-matched WT mice. b) Thy-tau22 transgenic model presented an age-dependent phospho-tau accumulation principally in the CA1 region, determined by AT8 immunohistochemistry (b1) or western-blots (b2). This phospho-tau accumulation produced an attenuated microglial response as probed by Iba1 immunostaining, from 12-16-month-old WT (b2) and Thy-tau22 (b3) mice. Microglial activation was also assessed by qPCR (b5). Only the expression of CD45, CD68 and TREM2 was slightly but significantly increased (ANOVA and Tukey post hoc test; *$p<0.05$). Data (n=5 animals per age) were normalized using 9-month-old WT mice (n=5; not shown) and are mean ± S.D. Quantitative analysis of the microglial domain (b6) demonstrated the existence of a significant decrease (Mann-Whitney U test; p=0.01; WT n= 75 or Thy-tau22 n=100 cells from 3 or 4 different animals, respectively) in the area covered by the individual microglia cells in aged (12-16-month-old) Thy1-tau22. DG, dentate gyrus; CA1-CA3 hippocampal subfields. Asterisks indicate amyloid

plaques. Scale bars: a1, a3 and a5, 500μm; a4 and a6, 20 μm; b1, 200 μm; b3 and b4, 20 μm

Supplemental Figure 4. Preparation and characterization of the S1 soluble fractions from Braak II, III-IV and V-VI hippocampus.

a) Hippocampal samples were fractionated into: S1, soluble fraction containing extracellular/cytosolic proteins; S2, intracellular vesiculated fraction; S3, aggregated proteins, releasable by SDS treatment and P3, highly-aggregated proteins, only releasable by SDS-Urea. The presence of phospho-tau species was analyzed by western blots using AT100 (a1) or AT8 (not shown). As expected, Braak III-IV and Braak V-VI samples showed a prominent phospho-tau accumulation (AT100 positive tau in the SDS releasable pool (S3). This is consistent with the presence of aggregated and phosphorylated tau protein, forming intracellular tangles. We also observed a clear accumulation of AT100 positive tau protein in the S1 soluble fraction (extracellular and/or cytosolic) from Braak V-VI samples. Total tau in the soluble S1 fractions was assessed by sandwich ELISA (a2).

b) Soluble Abeta present in S1 fractions was quantitatively analyzed by ELISA (b1) or dot-blots (b2-3) using the OC antibody.

Supplemental Figure 5. Sarkosyl-insoluble phospho-tau was not toxic for microglial cells and astrocytes were resistant to soluble phospho-tau.

a) Representative AT8 western blot of sarkosyl-soluble and sarkosyl-insoluble tau, isolated from two different Braak II and Braak V-VI samples (a1). The toxicity on BV2 cells of the sarkosyl-insoluble phospho-tau was then tested by Flow Cytometry (a2). Although we have assessed three different doses (0.1, 1 and 10 μl of sarkosyl-insluble tau/ 100 μl culture media; equivalent to ten to a hundred times higher dose than S1), we did not detect any toxic effect on BV2 cells (a3). Data are mean ± SD of three independent experiments. b) Toxicity (using flow cytometry, b1) of S1 fractions isolated from Braak 0, Braak II, Braak III-IV and Braak V-VI samples was also assessed using the astrocytic cell line WJE. As shown (b2), no toxicity was observed in any case. Data are mean ± S.D. of three independent experiments.

Supplemental Figure 6. Characterization and toxicity of the S1 soluble fractions prepared from APP/PS1 and Thy-tau22 tansgenic models and SH-SY5Y-tau transfected cells.

a) S1 fractions, isolated from APP/PS1 mice, accumulated oligomeric soluble Abeta (detected by dot-blots using OC, a1). S1 fractions isolated from age-matched WT or Thy-tau22 mice (see below) were used as negative controls. a2) Quantitative analysis of BV2 cell toxicity after treatment with 6- or 18-month-old APP/PS1-derived S1 fractions showing percentage of viable, early apoptotic cell (Annexin V positive) or late apoptotic cells (Annexin V and PI positive cells). Data are mean ± S.D. of three different experiments. b) S1 fractions isolated from Thy1-tau22 models. The phospho-tau (b1, upper panel) or total tau content (b1, middle panel) in these fractions was assessed by western blots using AT8 or Tau46 antibodies. As shown, there was an age-dependent increase in the soluble AT8-positive phosho-tau in the S1 fractions. (b2) Quantitative analysis of BV2 cell viability after treatment with 2-, 9- or 12-month-old Thy1-tau22-derived S1 fractions. Percentage of viable, early apoptotic cell (Annexin V positive) or late apoptotic cells (Annexin V and PI positive cells) was indicated in the figure. Only S1 fractions derived from 12-month-old Thy-tau22 presented toxicity at the dose used (0.1µl/100µl culture media). Data are mean ± S.D. of three different experiments. c) The presence of soluble phospho-tau in transfected SH-tau cells was also assessed. For these experiments, the cells were disrupted by sonication and the soluble and insoluble fractions isolated by centrifugation at 30,000xg (30 min at 4ºC). Total proteins were isolated using Laemmli-dissociation buffer. As shown, most tau forms (either phosphorylated, AT8-positive, or total, Tau46-positive) were isolated in the soluble fraction. This experiment was repeated twice with similar results.

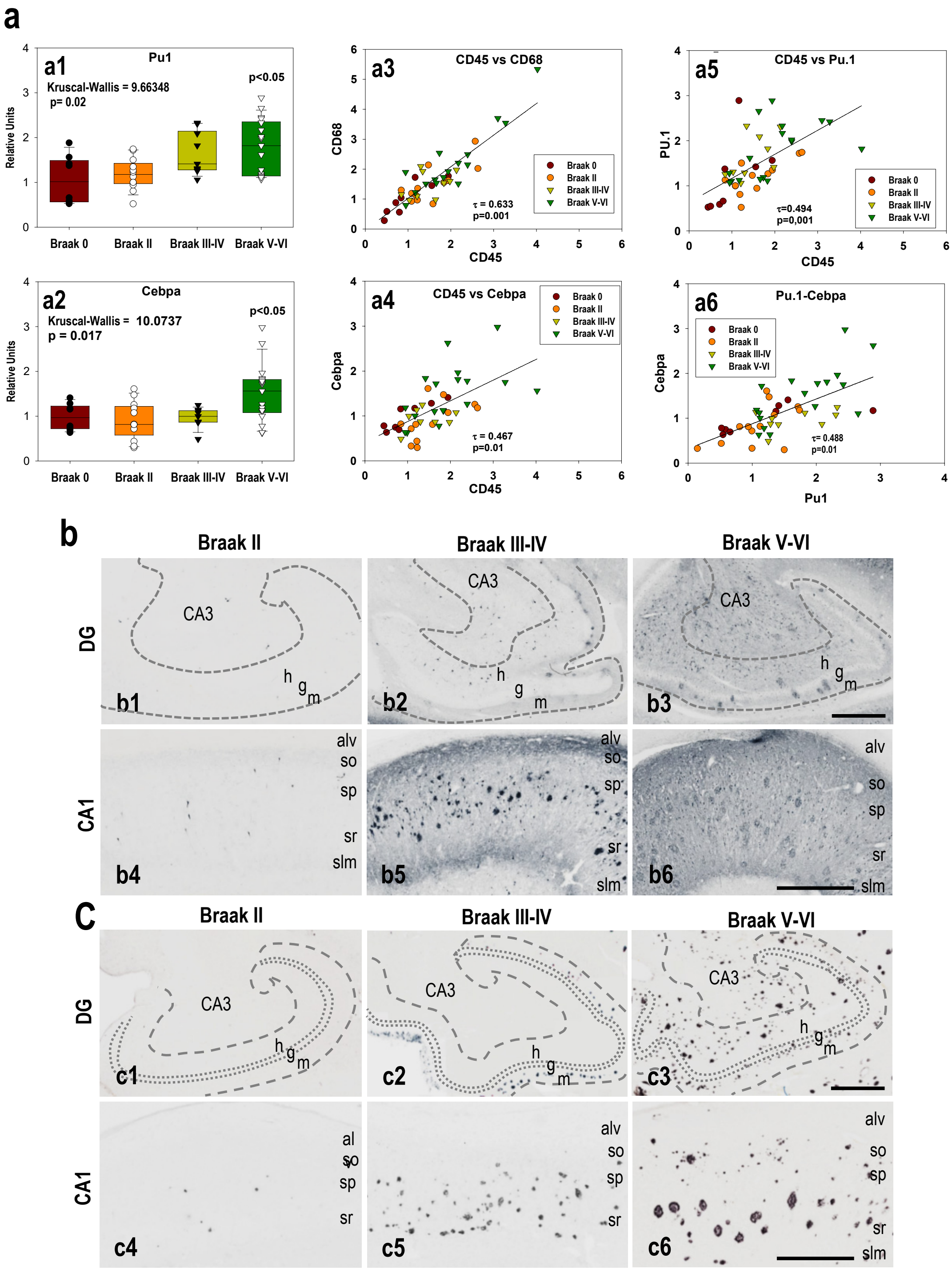

Supplemental Fig. 1

a

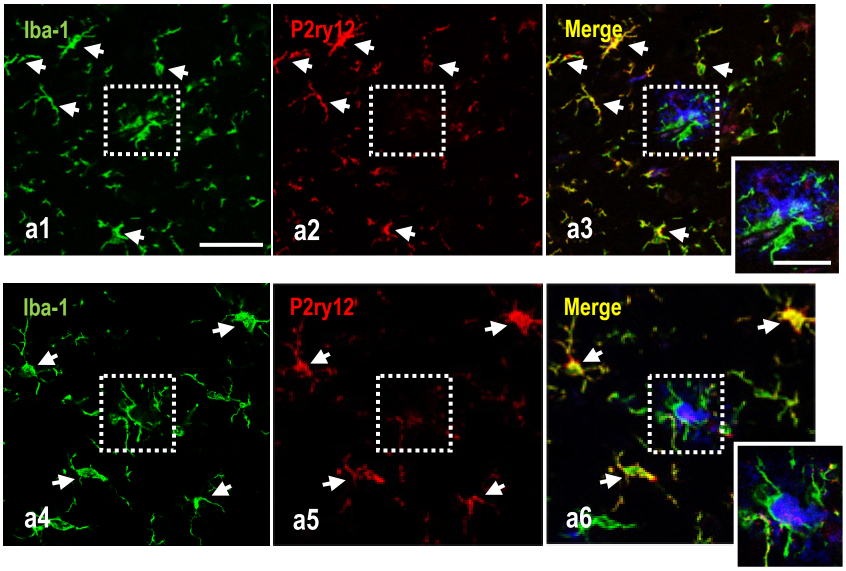

Supplemental Fig. 2

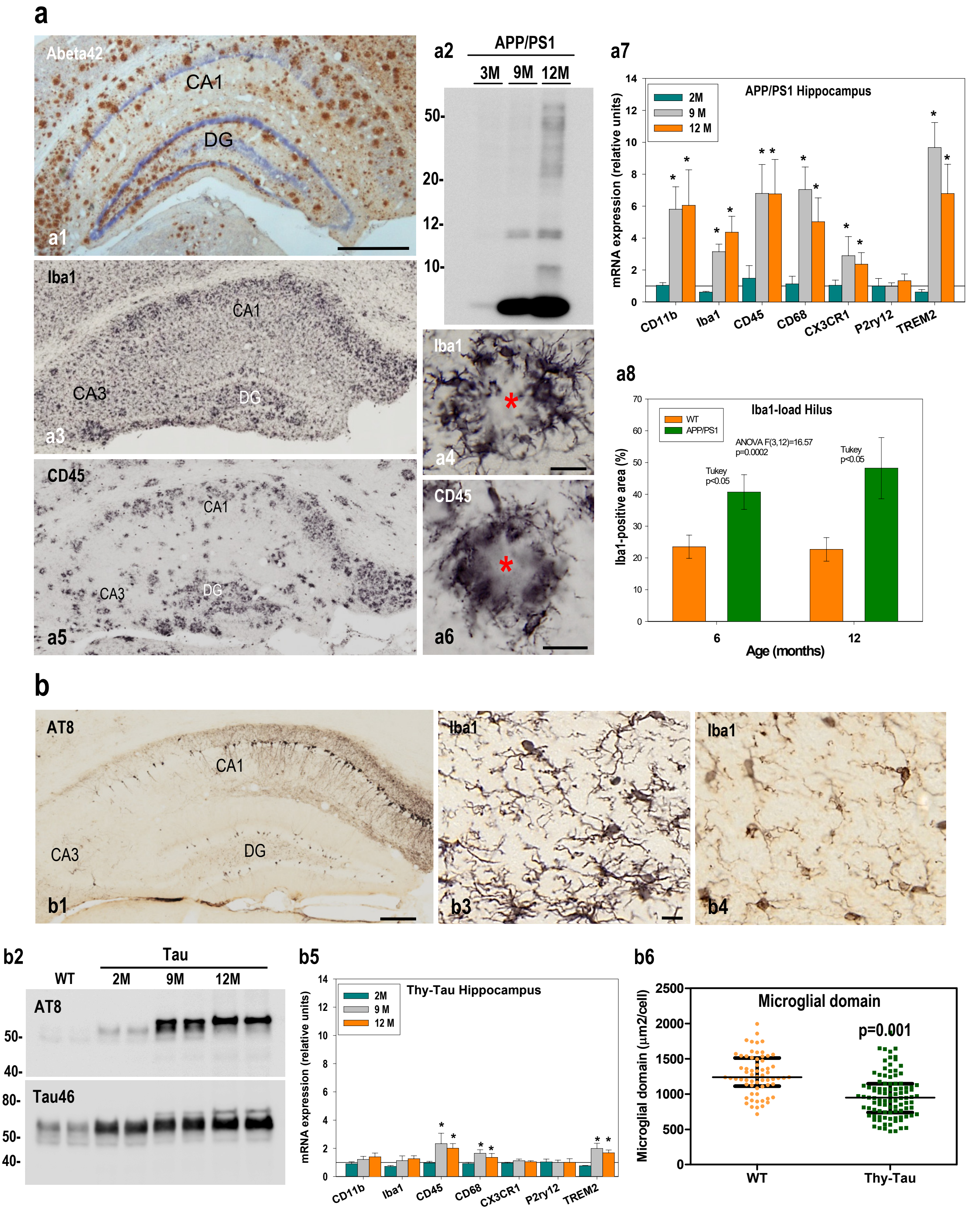

Supplemental Fig. 3

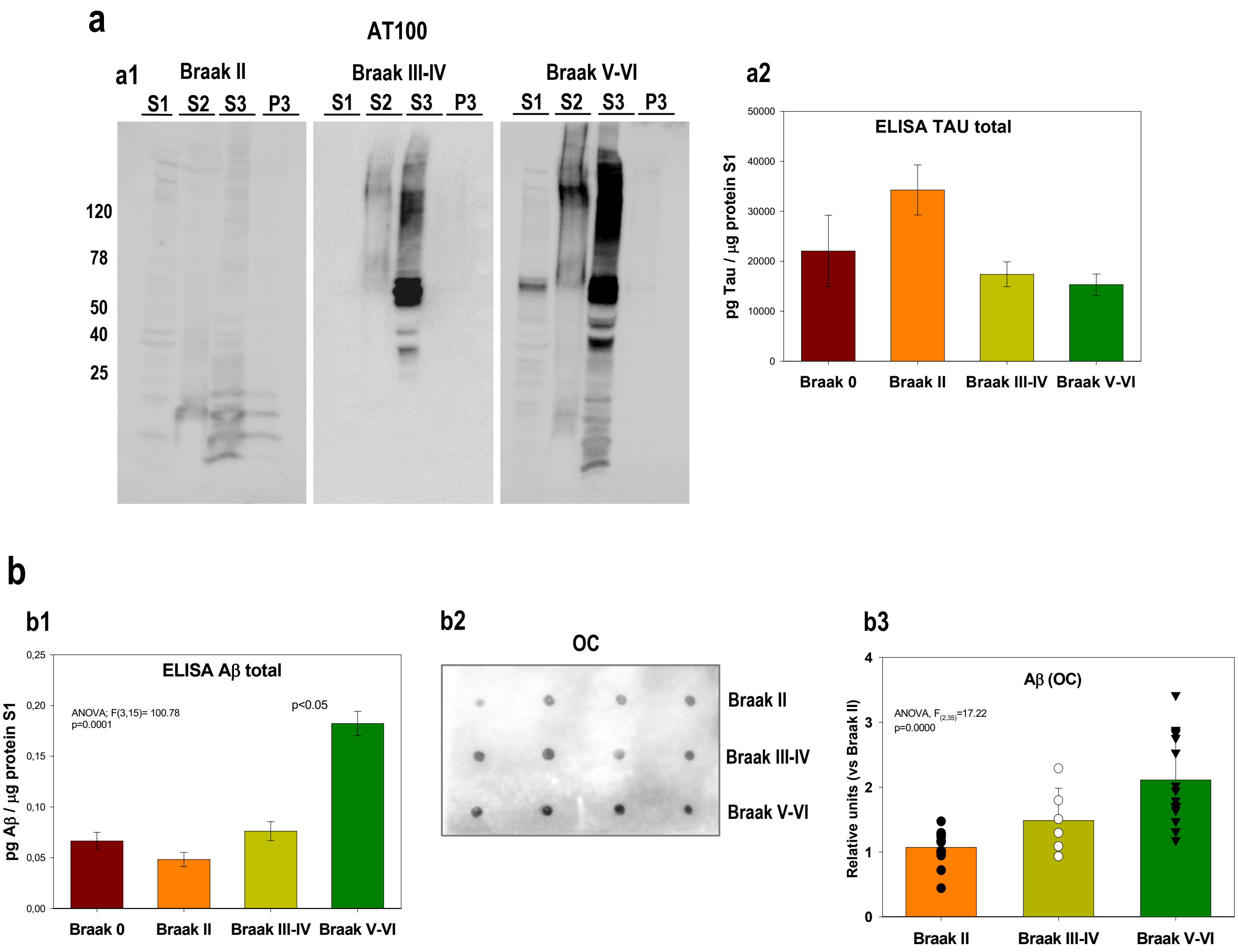

Supplemental Fig. 4

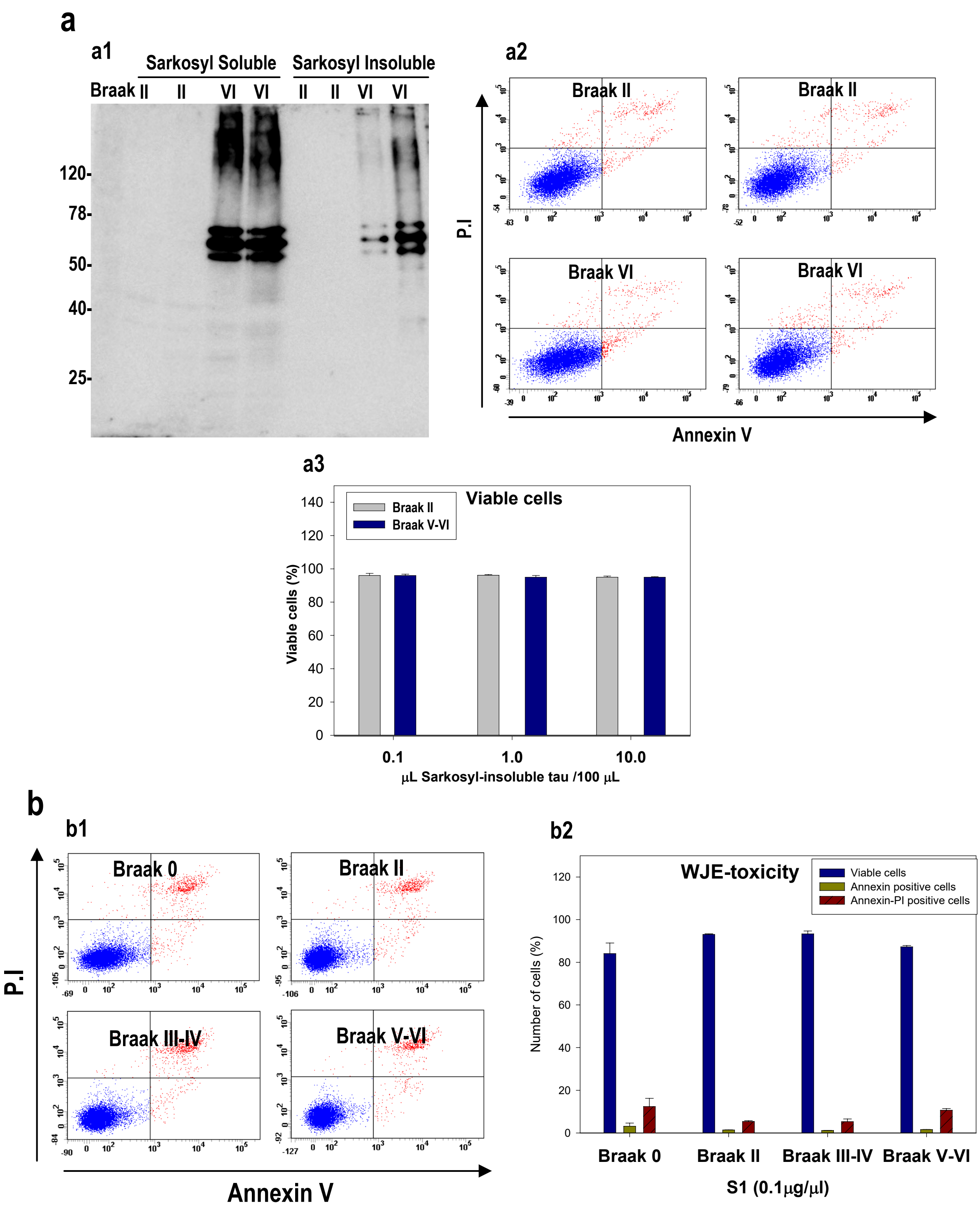

Supplemental Fig.5

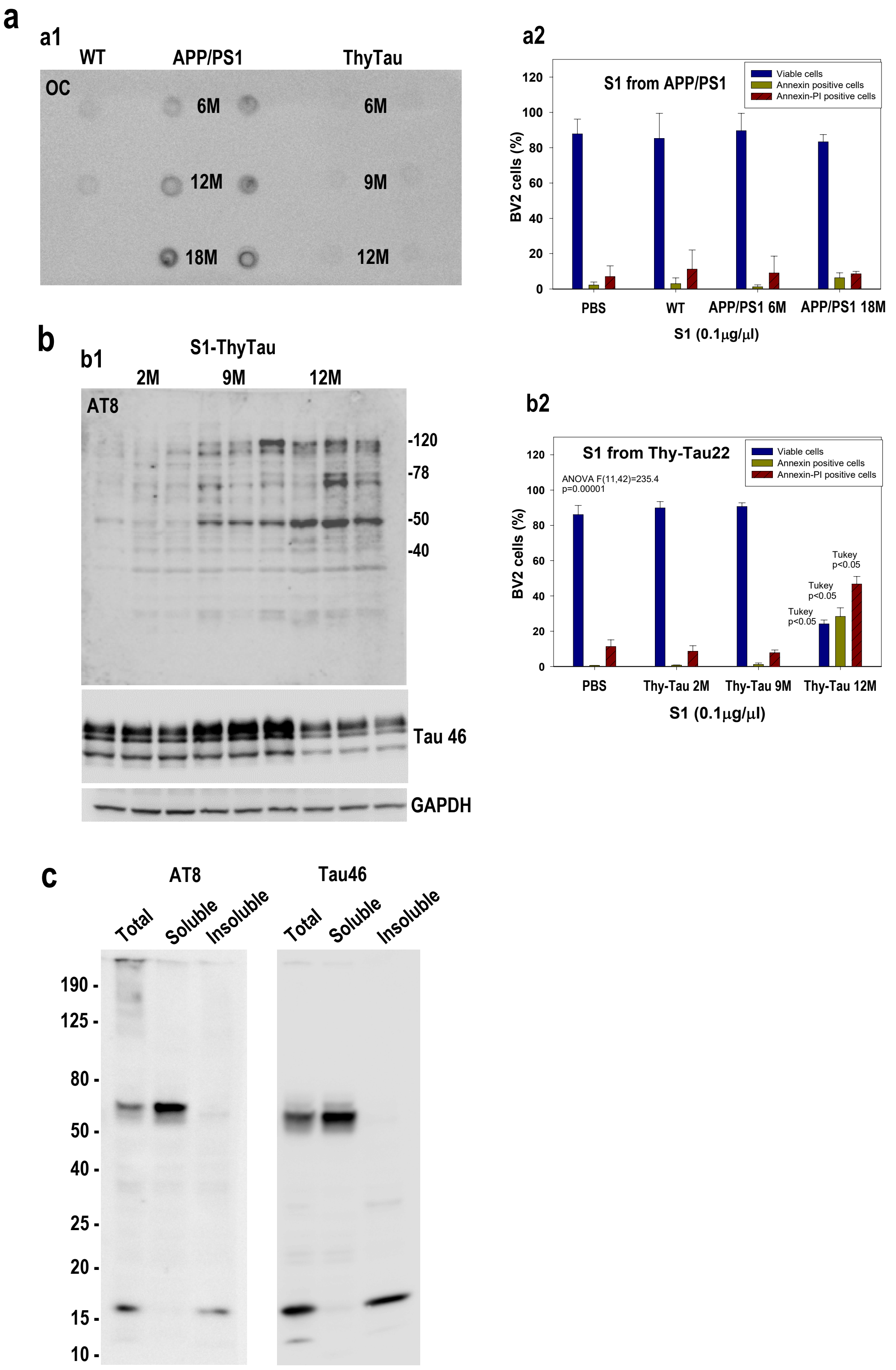

Supplemental Fig.6
